# Differential Expression of Exosomal microRNAs in Prefrontal Cortices of Schizophrenia and Bipolar Disorder Patients

**DOI:** 10.1371/journal.pone.0048814

**Published:** 2013-01-30

**Authors:** Meredith G. Banigan, Patricia F. Kao, James A. Kozubek, Ashley R. Winslow, Juan Medina, Joan Costa, Andrea Schmitt, Anja Schneider, Howard Cabral, Ozge Cagsal-Getkin, Charles R. Vanderburg, Ivana Delalle

**Affiliations:** 1 Department of Pathology and Laboratory Medicine, Boston University School of Medicine, Boston, Massachusetts, United States of America; 2 Advanced Tissue Resource Center, Harvard NeuroDiscovery Center, Charlestown, Massachusetts, United States of America; 3 Department of Biomedical Genetics, Boston University School of Medicine, Boston, Massachusetts, United States of America; 4 Department of Neurology, Massachusetts General Hospital, Harvard Medical School, Charlestown, Massachusetts, United States of America; 5 Neurological Tissue Bank, Parc Sanitari Sant Joan de Deu, Sant Boi de Llobregat, Barcelona, Spain; 6 Department of Psychiatry, Ludwig Maximilian University, Munich, Germany; 7 Laboratory of Neuroscience, Department and Institute of Psychiatry, Faculty of Medicine, University of São Paulo, São Paulo, Brazil; 8 Department of Psychiatry, University Medicine Goettingen and German Research Center for Neurodegenerative Diseases, Goettingen, Germany; 9 Department of Biostatistics, Boston University School of Public Health and Boston University Clinical and Translational Science Institute, Massachusetts, United States of America; University of Illinois at Chicago, United States of America

## Abstract

Exosomes are cellular secretory vesicles containing microRNAs (miRNAs). Once secreted, exosomes are able to attach to recipient cells and release miRNAs potentially modulating the function of the recipient cell. We hypothesized that exosomal miRNA expression in brains of patients diagnosed with schizophrenia (SZ) and bipolar disorder (BD) might differ from controls, reflecting either disease-specific or common aberrations in SZ and BD patients. The sources of the analyzed samples included McLean 66 Cohort Collection (Harvard Brain Tissue Resource Center), BrainNet Europe II (BNE, a consortium of 18 brain banks across Europe) and Boston Medical Center (BMC). Exosomal miRNAs from frozen postmortem prefrontal cortices with well-preserved RNA were isolated and submitted to profiling by *Luminex* FLEXMAP 3D microfluidic device. Multiple statistical analyses of microarray data suggested that certain exosomal miRNAs were differentially expressed in SZ and BD subjects in comparison to controls. RT-PCR validation confirmed that two miRNAs, miR-497 in SZ samples and miR-29c in BD samples, have significantly increased expression when compared to control samples. These results warrant future studies to evaluate the potential of exosome-derived miRNAs to serve as biomarkers of SZ and BD.

## Introduction

Knowing which molecules are specifically altered in neuropsychiatric patients would represent a crucial step towards uncovering mechanisms of the development of neuropshychiatric diseases and generating more successful therapeutic strategies. An immediate impact of identifying such molecules would be access to a set of biomarkers to help define and monitor populations at risk.

MicroRNAs (miRNAs, miRs) may up- or down-regulate the translation of messenger RNA (mRNA) or render it unstable, and have recently been proposed as biomarkers for brain neoplasms, degenerative diseases, autism, and schizophrenia [1]. Dysregulation of miRNAs in brains of patients diagnosed with schizophrenia (SZ) [2] and other neuropsychiatric disorders is plausible considering that many miRNAs are expressed in human brain [3] where they regulate neuronal development [4] and differentiation [5] including dendritic spine development [6] and plasticity [7],[8], as well as cognitive functions [9]. A group of miRNAs has been recently designated as “misexpressed” in the prefrontal cortices (PFCs) of both SZ and bipolar disorder (BD) patients [10]. The target analysis of another reported set of differentially expressed miRNAs in the PFCs of SZ patients revealed many genes implicated in signaling pathways [11]. While a specific BD miRNA profile has not yet been established, alterations in neurochemical regulation including an excess in signaling activity have been associated with BD [12].

Signaling activity in neurons commonly requires the release of signaling molecules from their specific secretory vesicles [13]. Exosomes are secretory vesicles defined by size (30–90 nm), buoyant density (∼1.1–1.2 g/ml), lipid composition, and the presence as well as the absence of specific marker proteins [14]. Exosomes represent the alternative route for the content of multivesicular bodies (MVBs, exosomal precursor organelles) destined for degradation in lyzosomes. Rather then being degraded, MVBs’ intraluminal vesicles are fused with the plasma membrane and secreted into the extracellular space as exosomes [15]. Because of the specific molecules on their surface, including cell-adhesion proteins, exosomes can be incorporated by specific recipient cells [15],[16]. The exosomal involvement in neuronal signaling was suggested by the presence of MVBs in dendritic spines [17],[18] and by the resemblance of synaptic spinules (evaginations of the postsynaptic membrane that bud into presynaptic axon) to exosomes [19],[20]. The role of exosomes in synaptic activity is further corroborated by the dependence of long term potentiation (LTP)-induced structural spine plasticity on exocytic trafficking from recycling endosomes [21].

Cultured primary cortical neurons and astrocytes do release exosomes [22]. Studies of exosomal cargo in neurons and microglia have revealed proteins important for the development of neurodegenerative diseases such as Alzheimer’s disease [23]–[26] and Parkinson’s disease [27]. Exosomes in neuronal cell lines have also been shown to transfer prions [28] as well as wild-type and mutant superoxide dismutase, thus propagating cell-to-cell mutant toxicity [29]. Microarray analysis of exosomal content from mouse bone marrow mast cells and human and mouse mast cell lines has established the presence of mRNA and miRNA [30]. A new name for this mRNA - “exosomal shuttle RNA” [30] - has been proposed to underscore the ability of exosomes to mediate the exchange of genetic material between cells. Changes in exosomal miRNAs have been reported in patients diagnosed with Alzheimer’s disease [31], while RNA content from the glioblastoma microvesicles was shown to provide diagnostic biomarkers [32]. Postmortem human PFC samples have been used to successfully generate miRNA profiles [11]. Here we report the results of exosomal miRNA expression analysis in PFCs from patients diagnosed with SZ, BD, and mental illness-free controls. We have observed aberrations in SZ and BD samples in comparison to controls, suggesting both common and differential pathobiology. These results may forecast reproducible, disease-associated changes of miRNA expression serving as biomarkers in exosomes, cellular particles potentially obtainable from the cerebrospinal fluid (CSF) of living patients.

## Materials and Methods

### Human Brain Tissue

Written informed consent was obtained from the next of kin for the use of the postmortem brain tissue by the following sources of frozen PFC (Brodmann area 9, BA9): BrainNet Europe II, a consortium of 18 brain banks across Europe (BNE; cases SZ 1–6, BD 5–9, and C 6–10), McLean 66 Cohort Collection at Harvard Brain Tissue Resource Center (McLean; cases SZ 7 and 8, BD 1–4, and C 1–5), and autopsy service at Boston Medical Center (BMC; cases C 11–13) (Table 1).

**Table 1:** Analyzed human prefrontal cortices (BA9).

Case andDisease	Age	Gender/Hemisphere	PMI	Neuropathology and pertinentclinical history	Source	RIN	Experiment
SZ1	74	M/left	6	BB II, alpha-synuclein immunoreactivityin few SN neurons	BNE	8.3	L, P
SZ2	65	M/left	3.5	BB I, lacunar infarct in the caudate	BNE	8.3	L, P
SZ3	82	M/left	4.5	BB VI, amyloid angiopathy, lacunarinfarct in putamen	BNE	9.1	L, P
SZ4	77	M/left	6	BB II, old infarct in the frontal lobe	BNE	8.3	L, P
SZ5	84	M/left	2.5	BB II, amyloid angiopathy, argirofilicgrain disease	BNE	8.3	L, P
SZ6	79	M/left	2.5	multiple infarcts in the teritories of middleand posterior cerebral artery circulation	BNE	7.8	L, P
SZ7	63	M/right	22.3	BB I	McLean	7.6	L
SZ8	35	M/left	28	BB I, non-specific fronto-temporo-parietaland thalamic atrophy	McLean	6.1	L
BD1	40	M/left	30.8	acute hypoxic changes, focalarteriolosclerosis	McLean	6.7	L
BD2	38	M/left	22	parietal cortex infarct, focal acute hypoxicchanges, focal arteriolosclerosis	McLean	7.9	L
BD3	50	M/right	30.5	no neuropathology reported	McLean	7.1	L
BD4	74	M/right	25	no neuropathology reported	McLean	6.6	L
BD5	73	M/right	5.3	BB II, temporal SPs, small old corticaland striatal infarcts	BNE	6.2	L, P
BD6	70	M/left	6.4	BB III, many SP, few NP, contusions (dueto fall); alcohol abuse	BNE	6.2	L, P
BD7	68	M/left	NA	BB I	BNE	5.5	P
BD8	93	M/left	6	BB I	BNE	5.1	P
BD9	90	F/left	6.5	BB I-II, rare SP	BNE	7.7	P
C1	67	M/right	22.3	BB I, lacunar infarcts in putamen, focalarteriolosclerosis	McLean	8.0	L
C2	37	M/right	18.7	minimal acute hypoxic-ischemic changein hippocampus	McLean	6.4	L
C3	38	M/left	28.8	no neuropathology reported	McLean	7.7	L
C4	50	M/left	24.1	BB I	McLean	6.2	L
C5	40	M/right	28	no neuropathology reported	McLean	7.1	L
C6	68	M/left	10.2	no neuropathology reported	BNE	6.4	L, P
C7	71	M/left	7.6	BB I, lacunar infarct in internal capsule	BNE	6.1	P
C8	82	F/left	7	severe atherosclerosis in the circle ofWillis, focal gliosis in striatum	BNE	6.1	P
C9	96	M/right	5.4	BB I, rare SP, rare NP, hypoxic-ischemicchanges in striatum and hippocampus	BNE	6.0	P
C10	65	F/left	12.8	no neuropathology	BNE	8.1	P
C11	80	M/left	17	no neuropathology	BMC	5.9	P
C12	72	F/left	23	no neuropathology	BMC	5.1	P
C13	68	F/left	25	no neuropathology	BMC	7.0	P

SZ =  schizophrenia; BD =  bipolar disorder; C =  controls.

BB =  Braak and Braak.

L =  Luminex; P =  qPCR.

### Extractions of Exosome-containing Pellets

Exosome extraction protocol from primary and cultured cells [30] was modified to obtain exosome-containing pellets from frozen brain tissue that was previously evaluated for RNA integrity (Figure S1). Only the brain tissue samples yielding an RNA integrity number (RIN) of ≥5.1 (range 5.1–9.1, Table 1) and thus well within accepted standards [33] were used to obtain exosome-containing pellets. From the original frozen prefrontal cortex stored at −80°C procured by each brain bank, we cut approximately 600 mg of grey matter using a razor blade. The frozen tissue was put in 1 ml phosphate-buffered saline, pH 7.4 (PBS, 4°C), gently teased with a small spatula, and vortexed (VWR mini-vortexer) on a medium power. Each tube was centrifuged at 300×g for 10 min, decanted, centrifuged twice at 1,200×g for 10 min with decanting in between, and finally filtered through a 0.2 um syringe filter (Millipore, Carrigtowhill, Cork, Ireland). The filtrate was centrifuged twice at 10,000×g for 30 min with decanting in between. The final centrifugation to obtain exosome-containing pellets was performed at 22,000×g for 22 hrs. All centrifugations were performed at 2°C.

As a control exosomal extraction procedure, exosomes from cultured human H4 cells (HTB 148 ATCC, Manassas, VA, USA) were prepared as previously published [16]–[34] with minor modifications. Briefly, H4 cells were cultured in OPTIMEM medium (Life Technologies) without serum or antibiotics. After 48 hours, conditioned medium was collected and centrifuged for 5 min at 500×g at 4°C to remove cell debris. The supernatants were then sequentially centrifuged at 300×g (10 min) and twice at 2,000×g (10 min), 4°C. The supernatant was filtered through a 0.45 um (Whatman, Florham Park, NJ) and 0.22 um (Millipore, Carrigtowhill, Cork, Ireland) filter, and centrifuged for 1 hr at 10,000×g at 4°C. The supernatant was removed and then subjected to ultracentrifugation at 100,000×g for 2 hours at 4°C. The supernatant was collected and the exosome-containing pellet was re-suspended in PBS for Western Blotting.

### Transmission Electron Microscopy (TEM) and Immuno-gold Labeling

For morphological identification of exosomes, the pellets were either embedded in a hydrophilic resin upon fixation in TEM fixative (4% paraformaldehyde, 0.2% glutaraldehyde, in 0.2 M cacodylate buffer, pH 7.2.) or directly adsorbed to a carbon-coated grid that has been made hydrophilic by an exposure to a glow discharge (30 sec). Upon blocking with 1% Bovine Serum Albumin (BSA), grids with pellets were incubated in primary antibody (CD63, *BD Pharmingen*, and GAPDH, *Ambion*) solutions in 1% BSA, rinsed in PBS, exposed to either bridging antibody first or directly to protein A-gold (5 nM) solution in 1% BSA, rinsed in PBS, and finally in water. Excess liquid was removed with a filter-paper (Whatman #1). Optional negative staining was achieved by incubation in 0.75% uranyl formate for 30 seconds. The examination was carried out in a JEOL 1200EX TEM or a TecnaiG^2^ Spirit BioTWIN and images were recorded with an *AMT 2k CCD* camera.

### Western Blotting

The expression of an exosomal marker flotillin-2 [35] in the exosomal preparations from frozen postmortem PFC was compared to the flotillin-2 expression in the cell-culture-derived exosomal preparations described above. Equal protein amounts (2.5 mg/ml) from each exosome-containing pellet reconstituted in PBS and respective supernatant were blotted with anti-flotillin-2 antibody (*BD Transduction Laboratories*, 1∶1,000).

### Isolation, Purification, and Linear Amplification of miRNA from Exosome-containing Pellets

Exosome-containing pellets re-suspended in 20 ul of PBS were incubated with 0.4 mg/ml of RNase A/T1 (*Fementas*) for 10 min at 37°C. RNase was inactivated by adding 20 units/ul of *SuperRase-in* RNase inhibitor (*Ambion*) for 10 min at 25°C. The RNase treatment destroyed the higher molecular weight extra-exosomal cellular RNAs and preserved the small RNAs contained in the exosomes, i.e. ribosomal RNA and miRNA (Figure S2). Upon RNase inactivation, 60 ul of miRNA extraction buffer (*Arcturus*) was added, followed by an incubation at 42°C for 30 min and purification using *PicoPure* RNA Isolation Kit (*Lifetech*). The expected size range of miRNAs (∼19–28 nucleotides, nt) and pre-miRNAs (45–60 nt) were well-represented in the purified preparations (Figure S2). Because *Luminex* profiling system operates with inputs of 2.5–5 ug of total RNA per sample, we needed to ensure that our samples are sufficiently enriched for miRNA. To that end, we used *Invitrogen NCode* miRNA amplification system according to the manufacturer’s protocol to linearly amplify miRNA in our material (Table S2). We found that this amplification step upon above described RNA treatment did not induce changes in the RNA profile (Figure S3).

### Luminex miRNA Assay

We performed miRNA expression analysis using a FlexMAP3D instrument by (Luminex Corporation, Austin, TX) and a manufacturer’s assay for 312 miRNA (Table S1). This assay uses 5.6 um polystyrene micro-beads each of which contains a mixture of two fluorescent dyes that enable the beads to be identified as one of a specific set. Oligonucleotides, specific for each of our 312 miRNA assayed, were attached to the beads according to the manufacturer’s protocol. The beads, upon incubation with miRNA containing samples, were passed through a fluidic tube that causes the micro-spheres to line up in single file before they pass through the detection chamber that contains two lasers. One of the laser beams classifies each bead into the appropriate bead set, while the other scans the beads for the presence of fluorescent reporter molecules and quantifies the number of reporter molecules on each bead. All 312 miRNA were assayed simultaneously in each sample. Intra-normalization of the obtained expression values was performed according to the *Luminex* protocol. Negative values were considered “0” because they indicate the expression below the background, i.e. the absence of a specific miRNA signal (Table S1).

### Statistical Analysis of Luminex miRNA Expression Data

Student’s t-tests and statistical software packages including an *Excel* plug-in named Significance Analysis of Microarrays (SAM) [36],[37] and an *R* package named Prediction Analysis of Microarrays (PAM) [38] were used to analyze *Luminex* expression data on 312 miRNAs from the prefrontal cortex of 8 SZ, 6 BD, and 6 control samples (Table 1 and Table S1). Because the statistical analysis of these data involves multiple comparisons we applied Bonferroni Step-down Holm Correction [39] to Student’s t-test outcomes [0.05/(312–number of miRNAs ranked higher)], resulting in a stricter threshold for significance (Table S1). Next, we used SAM and PAM because they provide multiclass testing [36]–[38] while a simple t-test only enables analysis between two groups. Multiclass testing was a necessary tool to identify a set of miRNAs that could be used to differentiate between all three groups. Both SAM and PAM use a modified z-score statistic [36]–[38] to produce a ranked list of candidate biomarker miRNAs. The adjustment for codependency of expression was performed as local False Discovery Rate (FDR) [37], [40] presented in Tables 2 and 3. FDR is a method of permutation testing [41] that evaluates the risk of Type 1 error. Local FDR is an FDR assessment of not only a single miRNA, but also its “neighbors”, i.e. other closely ranked miRNAs. In this way, a co-dependency is accounted for. Cluster analysis [38] was applied to those miRNAs that had “0″ q-values in SAM (Tables 2 and 3). Clustering was carried out through use of 2*(1-cc) where cc equals a correlation between the cubed root of a single miRNA expression value for a sample and the average of cube-rooted values for all samples in the cluster it joins at that time point. The highest possible correlation between samples is 1 and the lowest is 0, resulting in a dendrogram that presents the clustered samples on a scale of 0 to 2. The dendrogram begins clustering miRNA with most similar expression patterns at the bottom of the graph and then additional miRNA are added to existing clusters as the graph is assembled vertically. In order to assess the predictive power of SAM-ranked miRNAs, the misclassification rate [38]–[42] was established as a means to classify clinical groups. A low misclassification rate indicates that a miRNA or a set of miRNAs is a reliable predictor of a group phenotype (a biomarker). Because of the limited number of cases available for analysis, we performed Wilcoxon test to supplement our findings (Table S3).

**Table 2:** SAM test of *Luminex* miRNA expression data for all 3 examined groups of cases: SZ, BD, and C.

miRNA	z-score:C	z-score:BD	z-score: SZ	q-value(%)	local FDR(%)
hsa-miR-31	−2.19709	−2.54002	3.55283	0	1.553882119
hsa-miR-33	−2.17637	−1.95758	3.10046	0	2.433615922
hsa-miR-96	−1.99363	−1.9829	2.9824	0	2.493468788
hsa-miR-28	−1.40723	−1.68092	2.31611	0	2.300536436
hsa-miR-30e-5p	−1.01353	−2.15639	2.37744	0	2.438595672
hsa-miR-199a*	−1.52064	−1.43723	2.21841	0	2.580013124
hsa-miR-501	2.162398	−0.31372	−1.38651	0	2.585758655
hsa-miR-504	1.9952	−0.30377	−1.26857	0	2.662187029
hsa-miR-15b	−0.83294	−1.75496	1.94092	0	2.659473233
hsa-miR-29c	2.068676	−0.39402	−1.25599	0	2.649467758
hsa-miR-455	−1.49405	−1.08385	1.93342	0	2.611617439
hsa-miR-380-3p	1.878938	−0.83204	−0.78517	0	2.621226767
hsa-miR-323	−1.39982	−0.99834	1.79862	0	2.626487216
hsa-miR-527	1.535186	0.010721	−1.15943	0	2.807651873
hsa-miR-93	−1.31706	−0.81606	1.59984	0	2.851798957
hsa-miR-32	−1.01365	−1.26054	1.70564	0	2.86415899
hsa-miR-20b	−1.24865	−1.25458	1.87742	0	2.873400151
hsa-miR-516-5p	1.441361	−0.31318	−0.84614	0	2.997241768
hsa-miR-92	−1.12108	−0.96419	1.56396	0	3.013501643
hsa-miR-30a-3p	−1.18983	−1.04924	1.67931	0	3.063908167
hsa-miR-497	1.63309	−0.87672	−0.56728	0	3.16010056
hsa-miR-498	1.256994	−0.11349	−0.85762	2.17948718	4.322678597
hsa-miR-133b	−1.34128	−0.7324	1.55526	2.17948718	4.639798252
hsa-miR-499	1.736963	−0.88259	−0.64078	2.17948718	4.738725328
hsa-miR-10b	−0.72213	−1.17673	1.42415	2.17948718	4.878212949
hsa-miR-202*	−0.9101	−1.73414	1.98318	2.17948718	4.898293562
hsa-miR-202	−0.14303	1.238359	−0.82149	2.17948718	5.065305767
hsa-miR-149	1.490285	−0.74638	−0.55793	2.17948718	7.055913859
hsa-miR-523	0.88386	0.910065	−1.34544	2.17948718	7.62760309
hsa-miR-199b	−0.87254	−0.91723	1.34233	2.17948718	7.857837003
hsa-miR-377	−0.97987	−1.24239	1.6667	3.73626374	9.314491623
hsa-miR-518b	0.322664	0.980094	−0.97707	3.73626374	9.367786992
hsa-miR-26b	1.336505	−0.36918	−0.72549	3.73626374	9.819393964
hsa-miR-190	−1.44232	−1.32539	2.07578	3.73626374	10.24724163
hsa-miR-326	−0.1038	1.199379	−0.82169	3.73626374	10.34765218
hsa-miR-494	0.293894	0.910858	−0.90356	3.73626374	11.67285654
hsa-miR-30e-3p	1.314221	−0.87488	−0.32951	3.73626374	12.18718064
hsa-miR-512-3p	−0.17555	1.286875	−0.83349	3.73626374	12.89683212
hsa-miR-302a*	−0.47638	−1.30373	1.33509	3.73626374	13.04097715
hsa-miR-19b	−0.28889	1.482134	−0.89494	3.73626374	13.11908036
hsa-miR-302c	−0.12265	1.445789	−0.99236	4.84330484	14.25507383
hsa-miR-218	0.521858	1.207283	−1.29686	4.84330484	14.36727552
hsa-miR-338	−1.33167	−0.78432	1.58699	4.84330484	14.76902994
hsa-miR-423	0.477963	0.812936	−0.96817	4.84330484	14.83356578
hsa-miR-200c	−1.36615	−1.22514	1.94347	4.84330484	15.64595147
hsa-miR-325	0.109128	1.083419	−0.89441	4.84330484	15.97503816
hsa-miR-376b	1.029142	−0.00167	−0.7706	4.84330484	16.33334873
hsa-miR-518c	−1.34489	−0.8751	1.66499	4.84330484	17.02063785
hsa-miR-525*	−0.93878	−1.06791	1.50502	4.84330484	17.14036115
hsa-miR-125b	−0.86013	−0.85856	1.28902	4.84330484	17.17411684
hsa-miR-299-3p	1.138288	−0.02057	−0.83829	4.84330484	18.19059244
hsa-miR-363*	0.02007	0.987996	−0.75605	4.84330484	18.33451874
hsa-miR-542-3p	0.990978	0.007651	−0.74897	4.84330484	18.33773541
hsa-miR-371	0.798332	0.593946	−1.04421	4.84330484	18.36737645
hsa-miR-105	1.116132	0.542534	−1.244	7.00549451	22.95334699
hsa-miR-449	0.914034	0.511142	−1.06888	7.00549451	23.04808523
hsa-miR-370	1.088913	−0.34469	−0.55817	8.02968961	25.80170896
hsa-miR-302d	−0.82471	−1.17696	1.50126	8.02968961	27.03187735
hsa-miR-22	−1.01777	−1.05168	1.55209	8.02968961	27.64688383
hsa-miR-17-5p	0.337338	0.922478	−0.94486	8.02968961	27.75618225
hsa-miR-518a-2*	1.201014	−0.2914	−0.68221	8.02968961	28.15873897
hsa-miR-103	−0.48953	−0.73475	0.91821	8.02968961	28.42825986
hsa-miR-520d*	1.460088	−0.4613	−0.74909	9.90675991	29.0140902
hsa-miR-18a*	−0.89462	−0.66084	1.1666	9.90675991	30.34475207
hsa-miR-210	−0.05258	1.249962	−0.89804	9.90675991	31.08028153
hsa-miR-520e	−0.47633	−1.04849	1.14362	9.90675991	31.70287333
hsa-miR-516-3p	1.187797	0.108884	−0.97251	9.90675991	32.5639352
hsa-miR-329	−0.9053	1.414948	−0.38224	9.90675991	33.43751026
hsa-miR-182	−1.07408	−0.71552	1.3422	9.90675991	33.54506373
hsa-miR-380-5p	0.31583	0.983345	−0.97438	9.90675991	34.0190772
hsa-miR-30c	−1.18267	0.402311	0.58527	9.90675991	35.0288034
hsa-miR-106b	−0.08408	1.027975	−0.70792	11.587147	35.57300849
hsa-miR-219	1.517496	−0.96688	−0.41296	11.587147	35.95711034
hsa-miR-16	−0.92068	−0.50831	1.07174	11.587147	36.48103455
hsa-miR-490	0.150144	0.835278	−0.73907	11.587147	37.01897704
hsa-miR-367	0.797077	0.41705	−0.9106	11.587147	37.31177035
hsa-miR-320	−0.25449	0.937805	−0.51249	11.587147	37.998358
hsa-miR-135b	0.337006	0.857104	−0.89558	11.587147	38.84789583
hsa-miR-206	−0.3697	0.94394	−0.43068	11.587147	39.10460163
hsa-miR-526b*	1.208306	−0.78313	−0.31888	11.587147	40.34787385
hsa-miR-425	−0.85102	1.009775	−0.11907	11.587147	40.77866903
hsa-miR-376a	0.920047	−0.1274	−0.59448	11.587147	40.92694087
hsa-miR-503	0.888295	−0.13607	−0.56417	14.1171329	42.85245703
hsa-miR-487a	0.162841	0.82095	−0.73784	14.1171329	43.30927731
hsa-miR-106a	0.521766	0.595635	−0.83805	14.1171329	43.48858371
hsa-miR-100	0.056388	−1.20747	0.86331	14.1171329	44.10398892
hsa-let-7f	−0.93439	−0.05581	0.74264	14.1171329	44.67888809
hsa-miR-511	0.310344	−1.0703	0.56996	14.1171329	44.90173075
hsa-miR-544	1.131482	−0.56382	−0.42574	14.1171329	47.12197196
hsa-miR-487b	−0.82112	−0.41659	0.92829	14.1171329	47.26591312
hsa-miR-363	−1.27178	−0.56825	1.38003	14.1171329	47.48233294
hsa-miR-376a*	0.390564	0.732719	−0.84246	14.1171329	48.56245152
hsa-miR-375	0.243853	0.644572	−0.66632	16.5048544	49.01224431
hsa-miR-153	−0.9969	−1.14696	1.60789	16.5048544	49.40211523
hsa-miR-431	−0.82106	−0.91197	1.29978	16.5048544	49.44548869
hsa-miR-217	−0.99016	−0.31608	0.97968	16.5048544	49.52370084
hsa-miR-362	−0.28573	−1.02521	0.98321	16.5048544	49.87006572
hsa-miR-500	0.980354	−0.56183	−0.31389	16.5048544	50.74516842
hsa-miR-372	−0.96458	−0.95534	1.43994	16.5048544	50.99446275
hsa-miR-148a	0.828874	−1.04752	0.16398	16.5048544	51.40114981
hsa-miR-10a	−0.64047	−0.52695	0.87556	16.5048544	52.40792839
hsa-miR-433	−0.22833	0.946138	−0.53835	16.5048544	52.46791499
hsa-miR-29b	0.620123	−1.14248	0.39177	16.5048544	52.72930619
hsa-miR-382	−0.23578	0.894415	−0.49398	16.5048544	53.59705171
hsa-miR-335	−0.3399	0.856229	−0.38725	16.5048544	53.75101383
hsa-miR-381	−0.81724	−0.5501	1.02551	16.5048544	54.38769193
hsa-miR-513	0.767999	0.015731	−0.5878	18.2605683	54.93952212
hsa-miR-328	0.233808	0.700291	−0.70057	18.2605683	55.70006535
hsa-miR-518e	−0.73833	−0.62201	1.02026	18.2605683	56.00690632
hsa-miR-301	0.120635	0.905541	−0.76963	18.2605683	56.02980543
hsa-miR-25	−0.66639	−0.6356	0.97649	18.2605683	56.32551846
hsa-miR-518d	0.155299	0.838491	−0.74534	19.8489011	58.23570838
hsa-miR-342	0.551634	−1.20248	0.48813	22.5464191	59.93768974
hsa-miR-521	−0.96506	−0.45523	1.06522	22.5464191	60.4907877
hsa-miR-520a	0.84235	−1.11148	0.20185	22.5464191	60.95727772
hsa-miR-409-5p	0.882507	−0.76108	−0.09107	22.5464191	61.30748083
hsa-miR-485-3p	0.386518	0.492904	−0.65957	24.0302433	62.62241634
hsa-miR-200a	0.092787	−0.91805	0.61894	25.0641026	63.41566145
hsa-miR-29a	0.482776	0.519518	−0.75172	25.0641026	63.78211013
hsa-miR-429	0.055713	0.792367	−0.63606	25.0641026	63.82820222
hsa-miR-125a	0.805608	−0.59199	−0.16021	28.4739454	65.48468447
hsa-miR-502	0.754098	−0.67512	−0.05923	28.4739454	65.82932368
hsa-miR-17-3p	−0.43522	−0.7476	0.88711	28.4739454	66.19614625
hsa-miR-148b	0.174623	−0.94181	0.57539	28.4739454	66.43951644
hsa-miR-368	0.743991	−0.88733	0.10751	28.4739454	67.12123684
hsa-miR-142-3p	−0.20516	−1.124	0.99687	31.1598558	67.73036369
hsa-miR-205	0.249306	0.845495	−0.8211	31.1598558	67.86858747
hsa-miR-373*	0.052207	0.60096	−0.48988	31.1598558	68.0705944
hsa-miR-517*	−0.63383	−0.14053	0.58077	32.4408284	68.51734889
hsa-miR-186	−0.35485	0.80983	−0.34124	32.4408284	68.75244349
hsa-miR-452*	−0.27916	−0.91071	0.8924	32.4408284	70.00183058
hsa-miR-181d	0.830156	−0.52455	−0.2292	32.4408284	70.12549652
hsa-miR-489	−0.56225	−0.14396	0.52966	32.4408284	70.13050269
hsa-miR-183	0.159174	0.686645	−0.63436	32.4408284	70.19307622
hsa-miR-30d	−0.43691	0.770014	−0.24983	32.4408284	70.23265424
hsa-miR-483	0.815477	−0.84234	0.02015	32.4408284	70.60907344
hsa-let-7a	−0.70813	0.463107	0.18377	32.4408284	70.71775515
hsa-miR-451	−1.23771	0.247949	0.74232	32.4408284	70.91507517
hsa-miR-221	−0.87556	−0.58425	1.09486	32.4408284	71.4883905
hsa-miR-507	−0.35197	−0.58369	0.70175	32.4408284	71.49526037
hsa-miR-526b	0.030514	0.581534	−0.45904	32.4408284	71.53582005
hsa-miR-496	0.850841	−0.05299	−0.59839	32.4408284	71.54645244
hsa-miR-185	0.031791	0.573309	−0.45383	32.4408284	71.56623333
hsa-miR-510	0.742406	−0.56788	−0.13089	32.4408284	71.60493642
hsa-miR-452	0.02098	0.573449	−0.44582	32.4408284	71.81598617
hsa-miR-127	−0.70529	−0.18458	0.6674	32.4408284	71.9211001
hsa-miR-212	−0.61587	0.689795	−0.05545	32.4408284	72.13105619
hsa-miR-26a	0.832095	-0.49388	−0.25366	32.4408284	72.20091674
hsa-miR-383	−0.45492	0.701135	−0.18466	32.4408284	72.32614427
hsa-miR-422a	−0.26395	0.806814	−0.40714	32.4408284	72.3764922
hsa-miR-518c*	0.001353	0.564767	−0.42459	41.3047974	72.53390646
hsa-miR-196b	−0.67575	0.967093	−0.2185	41.3047974	72.53717424
hsa-miR-150	0.050418	0.574385	−0.4686	41.3047974	72.67544996
hsa-miR-302b*	0.246155	−0.65687	0.30804	41.3047974	72.68497047
hsa-miR-134	0.023544	0.583001	−0.45491	41.3047974	72.76553336
hsa-miR-9*	0.6188	−0.93082	0.23401	41.3047974	72.77217111
hsa-miR-453	−0.74878	0.408178	0.25545	41.3047974	72.87972425
hsa-miR-34a	−0.58065	−0.27546	0.64208	41.3047974	72.96315149
hsa-miR-199a	−0.04344	0.698393	−0.49121	41.3047974	73.19135993
hsa-miR-526c	0.000846	0.548959	−0.41235	41.3047974	73.26518389
hsa-miR-515-3p	−0.73256	0.530075	0.15187	41.3047974	73.34759333
hsa-miR-215	−0.03675	0.717886	−0.51085	41.3047974	73.34812817
hsa-miR-181c	0.87805	−0.73324	−0.1086	41.3047974	73.58552846
hsa-miR-302c*	0.01393	0.540437	−0.41577	41.3047974	73.63892656
hsa-miR-211	−0.0302	0.714761	−0.51342	41.3047974	73.73914691
hsa-miR-515-5p	0.051513	0.532635	−0.43811	41.3047974	73.75924315
hsa-miR-520g	−0.95069	−0.92546	1.40711	41.3047974	74.09075907
hsa-miR-142-5p	−0.0799	0.677113	−0.44791	41.3047974	74.12613458
hsa-miR-524*	−0.00157	0.565716	−0.42311	41.3047974	74.15676194
hsa-miR-324-5p	−0.21206	0.663358	−0.33847	41.3047974	74.23525626
hsa-miR-432*	−0.13292	0.678476	−0.40916	41.3047974	74.28848132
hsa-miR-518f*	−0.03101	0.5454	−0.38579	41.3047974	74.30610926
hsa-miR-346	0.224671	0.480879	−0.52916	41.3047974	74.36735065
hsa-miR-216	−0.45903	0.7177	−0.194	41.3047974	74.44567177
hsa-miR-361	0.03535	0.671744	−0.53032	41.3047974	74.55343752
hsa-miR-98	−0.60393	0.234364	0.27717	41.3047974	74.60460599
hsa-miR-484	0.209461	0.738378	−0.71088	41.3047974	74.64404077
hsa-miR-189	−0.80392	−0.68433	1.11619	41.3047974	75.05309477
hsa-miR-126*	0.027436	0.621051	−0.48637	41.3047974	75.09259849
hsa-miR-545	0.351442	0.506327	−0.64333	41.3047974	75.16836638
hsa-miR-302a	−0.0864	0.685513	−0.44934	41.3047974	75.20719598
hsa-miR-432	−0.81081	0.255479	0.4165	41.3047974	75.25585407
hsa-miR-224	−0.65873	−0.04749	0.52966	41.3047974	75.292015
hsa-let-7i	−0.54415	−0.16195	0.52957	41.3047974	75.71872619
hsa-miR-137	0.070381	0.650415	−0.5406	41.3047974	75.76579176
hsa-miR-369-5p	0.749466	−0.27253	−0.3577	41.3047974	75.83285292
hsa-miR-526a	−0.04561	0.52662	−0.36076	43.4773989	76.60067761
hsa-miR-181b	0.487456	0.434359	−0.69136	43.4773989	76.6991424
hsa-miR-365	−0.08579	0.600065	−0.38571	43.4773989	76.70911636
hsa-miR-512-5p	0.584274	−0.53636	−0.03594	43.4773989	76.84890105
hsa-miR-493-5p	−0.39502	−0.55077	0.70934	43.4773989	77.04222504
hsa-miR-200a*	−0.41277	−0.31389	0.545	43.4773989	77.05673199
hsa-miR-509	0.610327	−0.27544	−0.25117	43.4773989	77.06179362
hsa-miR-187	−0.53083	−0.10011	0.4732	43.4773989	77.28591448
hsa-miR-214	−0.55444	0.28481	0.20223	43.4773989	77.6270228
hsa-miR-193b	−0.5695	−0.27523	0.63355	44.7455322	77.84340704
hsa-miR-18b	0.158782	−0.67872	0.38996	44.7455322	78.06910367
hsa-miR-27a	−0.67425	−0.14205	0.61222	44.7455322	78.15702062

The list of 198 miRNAs were narrowed from 312 microRNA using a linear regression model and subsequently ranked by z-score test statistics. Local FDR evaluates false discoveries by assigning samples to random groups to test for statistical significance by chance. The relevance of FDR is determined using q-values, an analog of the p-value. The 21 top-ranked miRNAs have a q-value equal to 0%, meaning that it is highly unlikely for this miRNAs to be expressed differentially by chance among the three groups examined.

SZ =  schizophrenia; BD =  bipolar disorder; C =  controls.

**Table 3:** SAM test of *Luminex* miRNA expression data for BD and C only.

miRNA	q-value(%)	local FDR(%)
hsa-miR-219	0	27.05850719
hsa-miR-380-3p	0	28.5520989
hsa-miR-499	0	29.39061044
hsa-miR-497	0	29.86048144
hsa-miR-149	0	30.16935542
hsa-miR-501	0	32.7806583
hsa-miR-29c	0	34.68749497
hsa-miR-30e-3p	12.10826211	36.00723604
hsa-miR-504	12.10826211	37.42550117
hsa-miR-148a	12.10826211	39.79413767
hsa-miR-520a	12.10826211	41.10693353
hsa-miR-526b*	12.10826211	43.98048662
hsa-miR-15b	17.20647773	47.26654182
hsa-miR-342	17.20647773	47.55108918
hsa-miR-29b	17.20647773	49.13804596
hsa-miR-409-5p	17.20647773	50.09111912
hsa-miR-516-5p	17.20647773	50.61439857
hsa-miR-520d*	17.20647773	50.79672442
hsa-miR-500	17.20647773	50.82589406
hsa-miR-368	18.95206243	53.22108834
hsa-miR-30e-5p	18.95206243	54.66652218
hsa-miR-181c	18.95206243	55.4595906
hsa-miR-26b	18.95206243	55.51339027
hsa-miR-511	22.70299145	58.90676274
hsa-miR-527	27.24358974	63.91589056
hsa-miR-483	27.24358974	64.697843
hsa-miR-544	27.24358974	65.0689104
hsa-miR-518a-2*	27.24358974	65.91311804
hsa-miR-370	30.06189213	67.72976336
hsa-miR-100	69.07664285	70.77603166
hsa-miR-498	69.07664285	71.22258291
hsa-miR-9*	71.07023411	74.68821123
hsa-miR-125a	71.07023411	76.54730008
hsa-miR-502	72.64957265	78.54613247
hsa-miR-26a	75.17482517	84.87201631
hsa-miR-181d	77.26224393	94.90250066
hsa-miR-510	77.26224393	95.66638517
hsa-miR-512-5p	78.16485434	100
hsa-miR-299-3p	78.16485434	100
hsa-miR-142-3p	78.16485434	100
hsa-miR-148b	82.00595701	100
hsa-miR-376a	82.00595701	100
hsa-miR-302a*	82.00595701	100
hsa-miR-503	82.00595701	100
hsa-miR-202*	82.00595701	100
hsa-miR-376b	82.00595701	100
hsa-miR-200a	84.15841584	100
hsa-miR-542-3p	84.15841584	100
hsa-miR-520e	84.15841584	100
hsa-miR-516-3p	84.15841584	100
hsa-miR-302b*	84.54907162	100

The results show 7 miRNAs with q-values equal to 0%.

### qPCR Analysis

Upon isolation and purification of RNA from exosome-containing pellets as above, the RNA concentration of each sample tested (Table 1) was calculated using *Agilent Bioanlyzer 2100* data. 20 ng of RNA was used to synthesize cDNA in a 20 ul reaction volume employing *Exiqon mIRCURY LNA* microRNA PCR Universal cDNA Synthesis Kit (Denmark). Quantitative PCR (qPCR) reactions for miRNAs of interest were performed according to the same manufacturer’s protocol. Briefly, cDNA was diluted to a final concentration of 0.1 ng/ul, added to a primer set generated by *Exiqon* and PCR SYBR Green Master Mix by the same manufacturer, and run for 40 cycles on a *Bio-Rad iCycler*. To obtain a change in CT (Delta-CT) for each miRNA of interest we needed to have an internal control in every sample. *Luminex* data as well as preliminary qPCRs established that miR-423 had a stable and robust expression in the samples tested (Table S1). Thus, all the analyzed samples were run in duplicates for each miRNA of interest and for miR-423 as a reference [43]–[45], (http://www.exiqon.com/ls/Documents/Scientific/miRNA-qPCR-guidelines.pdf) in a minimum of three separate plates. In each plate, the average CT value for miR-423 was subtracted from average CT value for a given miRNA of interest to obtain a value of change in CT (Delta-CT) for every sample. Student’s t-tests on control and BD and on control and SZ Delta-CT values were performed using *Graph Pad Prism 5*.

## Results

### Exosomal Marker Identification in Exosome-containing Pellets from Postmortem Human Prefrontal Cortex

**Figure 1:**
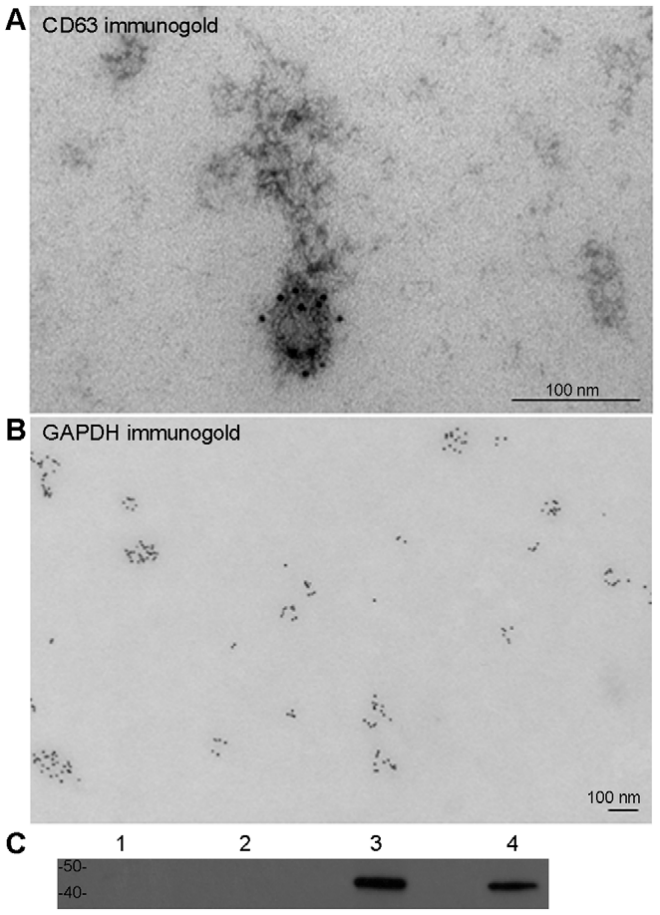
Characterization of exosome-containing pellets from human brain tissue. Electron microscopy of exosomal extractions from BA9 cortices demonstrates the presence of microvesicles (∼70–100 nm in diameter). Upon immuno-gold labeling procedure with antibodies against CD63 (A; additional negative staining highlights membrane of the vesicle) and GAPDH (B), the microvesicles reveal the presence of exosome-associated antigens. Bars indicate 100 nm. Comparison of exosomal extraction procedure products from BA9 cortices and H4 cell-culture reveals similar outcomes in Western blot. Supernatant of BA9 exosome-containing pellets (lane 1), supernatant of H4 exosome-containing pellets (lane 2), BA9 exosome-containing pellets reconstituted in PBS (lane 3), and of H4 exosome-containing pellets reconstituted in PBS (lane 4), show robust presence of exosomal marker flotillin-2 in the pellets, but not in the supernatants (C).

To confirm the presence of exosomes in our pellet-preparations from postmortem human frozen BA9 cortices, we examined the morphology and antigenicity of the pellet content (Figure 1). On electron microscopy, membrane-bound vesicles with diameters of 70–90 nm were immunoreactive for antigens commonly found in exosomes, CD63 and GAPDH (Figure 1A and B). Next, we evaluated the efficiency of our exosomal extractions from brain tissue. Western blot analysis revealed the presence of flotillin-2, protein commonly associated with exosomal membrane [35], in both BA9 pellets and in exosomes-containing pellets from the medium of cultured H4 cells (Figure 1C). The exosomal extraction procedures depleted flotillin-2 from the supernatants (Figure 1C).

### Luminex miRNA Expression Analysis in Exosome-containing Pellets from SZ and BD Patients Suggest Differential Expression of a Subset of miRNAs in Comparison to Controls

**Figure 2:**
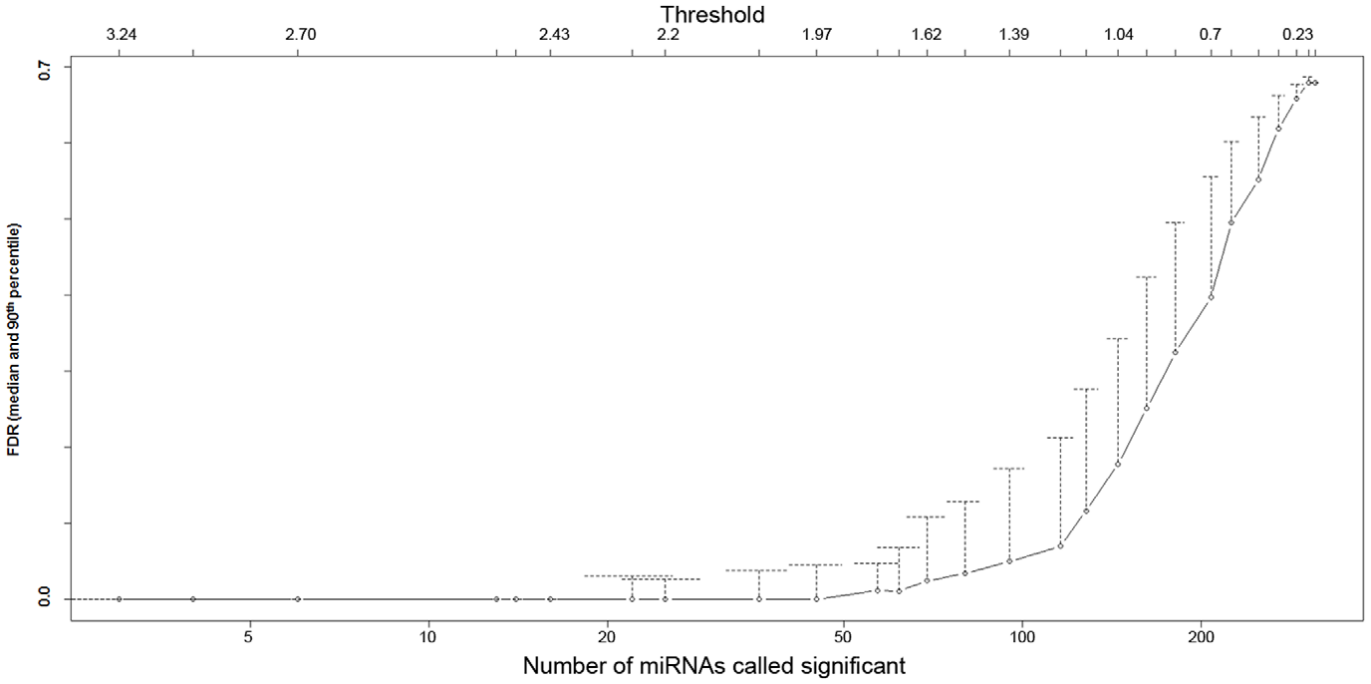
Median and 90th percentile of False Discovery Rates (FDR) in order left-to-right of threshold value and number of statistically significant results for all three groups (C, BD, and SZ). The Y-axis is an estimate of the percentage of false positives. High-ranked miRNA (at the left) have a low rate of false positives, while lower-ranked miRNA (moving toward the right) have higher rates of false positives.

**Figure 3:**
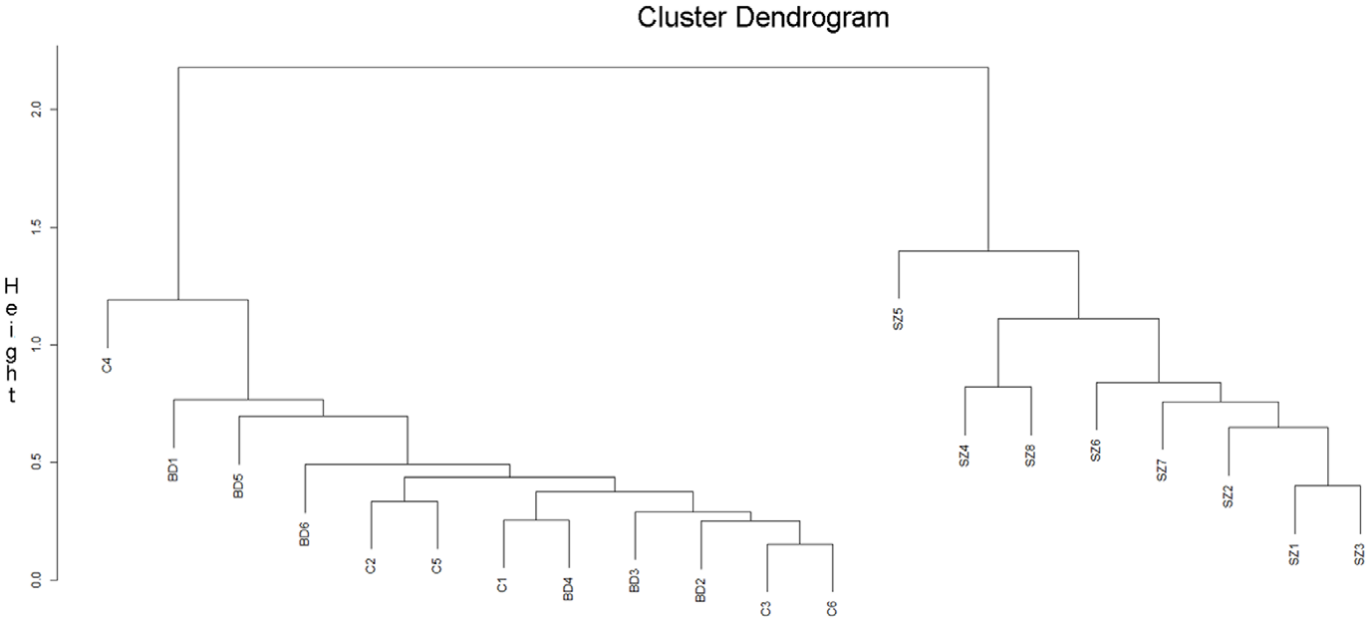
Hierarchical clustering analysis of top 21 ranked miRNAs from FDR analysis. Correlation coefficient (cc) was generated to assess the relationship between the expression values of each sample and the rest of the samples (see Methods). The coefficient is 1 if their expression profiles are highly similar and 0 if their expression profiles are highly divergent. The clustering graph is built so that the samples with similar expression patterns are clustered at the bottom while more differential patterns are at the top of the graph.

**Figure 4:**
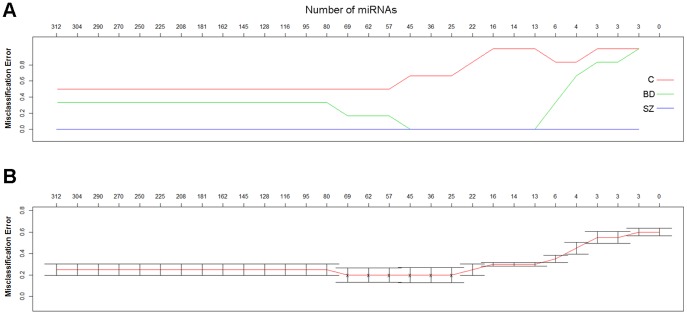
Misclassification rate analysis. (A) Controls (red) have the most variable miRNA expression. The expression data have less variability in BD (green) and the least in SZ (blue; misclassification rate  = 0), resulting in stronger predictive power within their respective clinical groups.

*Luminex* miRNA expression data in exosome-containing pellets from prefrontal (BA9) gray matter of 8 SZ patients (SZ 1–8), 6 BD patients (BD 1–6), and 6 controls (C 1–6) (Table 1) were submitted to Student’s t-test. These data reveal several significantly differentially expressed miRNAs in either BD or SZ, or both in comparison to controls. Bonferroni Step-down Holm Correction was then applied to correct for multiple comparisons [39]. This correction resulted in the absence of significantly differentially expressed miRNAs in BD samples, while the expression of only three miRNAs (miR- 31, -33, and -96) was significantly enhanced in SZ samples in comparison to controls and to BD (Table S1). In an alternative statistical approach towards identification of miRNAs differentially expressed in the three groups examined, we submitted raw *Luminex* miRNA expression data to Significance Analysis of Microarrays (SAM) program [36],[37] (Table 2). SAM ranked the expressions of examined miRNA expression using z-scores (Table 2). For the 21 top-ranked miRNAs, Prediction Analysis of Microarrays (PAM) [38]– derived q-value was 0% (Figure 2). We next examined how the expression of these 21 top-ranked miRNAs might influence the clustering of our samples. Surprisingly, the dendrogram from the clustering analysis [38] (Figure 3) suggests that the expression the 21 top-ranked miRNAs segregated SZ samples from BD samples and controls, leaving BD samples and controls undifferentiated from one another. In order to remove the influence of SZ group on the ranking system, the SAM program was run again for BD and control groups only (Table 3) and yielded 7 miRNAs with q- value of 0% (Table 3). This group of miRNAs, however, had a rather limited ability to segregate BD samples from controls (cluster dendrogram not shown). These results prompted us to submit the entire *Luminex* data to a PAM-derived misclassification rate analysis to determine if the miRNA expression values of each sample in a group have a reliable predictive power, i.e. if they can serve as biomarkers for a given group [42]–[38]. The misclassification rate was 0 for the SZ group (Figure 4), indicating that: a) the examined miRNA expressions were tightly grouped in SZ as previously indicated by the cluster dendrogram (see Figure 3); and b) the expression values of a very small number of miRNAs can assign an SZ sample to its correct group. The control samples had most variable miRNA expression levels associated with high misclassification rate regardless of how many miRNAs were evaluated (Figure 4). Finally, Figure 4 shows that the misclassification rate for BD samples is dependent on the number of miRNAs evaluated: if ∼13–16 miRNAs are taken into account, BD samples separate well from controls and reach zero misclassification rate that is characteristic of SZ samples (Figure 4).

### Covariate Analysis: the Effect of Medications on SAM Results

The clinical information accompanying our samples failed to provide sufficient data to control for tobacco, alcohol or recreational drug consumption (Table S4), while the information of prescribed medications (Table 4) was well defined. The cases were marked if the patients took at least one medication from the seven drug classes constructed on the basis of the common mechanism(s) underlying drug effect (A-G; Table 5). Since a standard error is used to generate a covariate effect, only drug classes with at least three case-subjects taking a drug and three case-subjects not taking a drug could be evaluated as covariates. Thus, only drug classes A (antipsychotics used in SZ only), B (neurotransmitter receptor-site binders used in both BD and SZ), and C (sedatives, hypnotics, anticonvulsants and analgesics used in both BD and SZ) could be controlled for.

**Table 4:** Medications (grouped in classes A-G) prescribed to subject cases for psychiatric and neurological symptoms.

Case	Medications prescribed for psychiatric and neurological symptoms, daily mg	A	B	C	D	E	F	G
SZ1	Levomepromazine 100, Haloperidol 10, Diazepam 5	X		X				
SZ2	Amisulpride 200, Quetiapine 200, Clonazepam 0.5, Oxcarbazepine 300		X	X				
SZ3	Haloperidol 5, Olanzapine 10, Lorazepam 1, Clomethiazole 192, Imipramine 50	X	X	X				
SZ4	Lormetazepam 1, Olanzapine 10, Lorazepam 1, Clomethiazole 192		X	X				
SZ5	Olanzapine 15		X					
SZ6	Phenitoine 100			X				
SZ7	Clozapine 400, Trazadone 50, Haloperidol 5, Lorazepam 1	X	X	X				X
SZ8	None							
BD1	Lithium 400, Methylphenidate 20, Diazepam 10, Oxazepam 20			X	X	X		
BD2	Lithium 400, Quetiapine 25, Midazolam (amount?)		X	X	X			
BD3	Risperidone 1.5, Gabapentin 600, Nefazodone 600, Topiramate 75, Ziprasidone 20		X	X			X	
BD4	Valproic acid 1500, Paroxetine 20, Clonazepam 4, Olanzapine 10		X	X			X	
BD5	Lithium (amount?)				X			
BD6	Valproic acid 1000, Quetiapine 125		X		X			
BD7	Moclobemide 300					X		
BD8	Dexamphetamine sulphate 5					X		
BD9	Tranylcypromine 60					X		
C1–C13	None							

SZ =  schizophrenia; BD =  bipolar disorder; C =  controls.

**Table 5:** Classes (A–G) encompassing the medications in Table 4.

Class	Description of medication class	Prescribed medicationfrom the class	Effects
A	Antipsychotics specifically for SZ	Levomepromazine	Phenothiazine Group; dopamine, adrenalin, histamine, acetylcholine and serotonin antagonist; antipsychotic effects
A	Antipsychotics specifically for SZ	Haloperidol	dopamine antagonist; effects similar to Phenothiazine group; antipsychotic effects
B	Neurotransmitter receptor- site binders usedin BD and SZ	Olanzapine	dopamine, serotonin, adrenergic antagonist with antihistaminic effect; antipsychotic properties
B	Neurotransmitter receptor- site binders usedin BD and SZ	Clozapine	dopamine, serotonin, adrenergic antagonist with antihistaminic effect; antipsychotic properties
B	Neurotransmitter receptor- site binders usedin BD and SZ	Quetiapine	dopamine, serotonin, adrenergic antagonist with antihistaminic effect; antipsychotic properties
B	Neurotransmitter receptor- site binders usedin BD and SZ	Amisulpride	dopamine and serotonin antagonist; antipsychotic properties
B	Neurotransmitter receptor- site binders usedin BD and SZ	Risperidone	dopamine, serotonin, adrenergic antagonist with antihistaminic effect; antipsychotic properties
B	Neurotransmitter receptor- site binders usedin BD and SZ	Ziprasidone	dopamine, serotonin, adrenergic antagonist with antihistaminic effect; antipsychotic properties
C	Sedatives, hypnotics, anticonvulsants and analgesicsin BD and SZ	Diazepam	Benzodiazipines, i.e. GABA agonists; sedative, hypnotic, with anticonvulsant effects
C	Sedatives, hypnotics, anticonvulsants and analgesicsin BD and SZ	Clonazepam	Benzodiazipines, i.e. GABA agonists; sedative, hypnotic, with anticonvulsant effects
C	Sedatives, hypnotics, anticonvulsants and analgesicsin BD and SZ	Lorazepam	Benzodiazipines, i.e. GABA agonists; sedative, hypnotic, with anticonvulsant effects
C	Sedatives, hypnotics, anticonvulsants and analgesicsin BD and SZ	Oxazepam	Benzodiazipines, i.e. GABA agonists; sedative, hypnotic, with anticonvulsant effects
C	Sedatives, hypnotics, anticonvulsants and analgesicsin BD and SZ	Midazolam	Benzodiazipines, i.e. GABA agonists; sedative, hypnotic, with anticonvulsant effects
C	Sedatives, hypnotics, anticonvulsants and analgesicsin BD and SZ	Clomethiazole	GABA agonist; sedative, hypnotic, with anticonvulsant effects
C	Sedatives, hypnotics, anticonvulsants and analgesicsin BD and SZ	Oxcarbazepin	ion, mostly sodium, channel stabilizer; anticonvulsant and mood stabilizing effects
C	Sedatives, hypnotics, anticonvulsants and analgesicsin BD and SZ	Phenitoin	sodium channel stabilizer, anticonvulsant effect
C	Sedatives, hypnotics, anticonvulsants and analgesicsin BD and SZ	Gabapentin	GABA agonist; anticonvulsant and analgesic effects
C	Sedatives, hypnotics, anticonvulsants and analgesicsin BD and SZ	Topiramate	mechanism unclear; anticonvulsant effect
D	Antipsychotics specifically for BD	Valproic acid	GABA effects enhancer; anticonvulsant and mood-stabilizing effects
D	Antipsychotics specifically for BD	Lithium	mechanism unclear; mood-stabilizing effects
E	Psycho-stimulants used in BD	Moclobemide	monoamine oxidase inhibitors; antidepressant effects
E	Psycho-stimulants used in BD	Tranylcypromine	monoamine oxidase inhibitors; antidepressant effects
E	Psycho-stimulants used in BD	Methylphenidate	dopamine enhancer, psychostimulant
E	Psycho-stimulants used in BD	Dexamphetamine	psychostimulant
F	Serotonin and adrenergic antagonists for BD	Nefazodone	serotonin and adrenergic antagonist; antidepressant effects
F	Serotonin and adrenergic antagonists for BD	Paroxetine	serotonin re-uptake inhibitor; antidepressant and anxiolytic effects
G	Serotonin antagonist for SZ	Trazodone	serotonin antagonist and reuptake inhibitor, antidepressant, anxiolytic, and sedative effects

SZ =  schizophrenia; BD =  bipolar disorder.

In a simple format of covariate adjustment [46] the effects of multiple independent variables are added together in a linear regression model. To guard against Type I errors, we designed a model to adjust for covariates using the SAM program. SAM was rerun to compare case subjects taking a specific drug class medication against case subjects not taking it, and a list of covariate effects for drug classes A–C was derived for top-ranked miRNA in the study (Table 6). Those effects were then subtracted from the original z-scores (Tables 2 and 3) and p-values were added to demonstrate the effects of the covariate on significance (Tables 7 and 8).

**Table 6:** Covariate effects of medications on highly-ranked miRNAs in SAM (top-21 in Table 2 and top-12 in Table 3).

miRNA	Class A	Class B	Class C
hsa-miR-31	−0.5474735	0.0027362	
hsa-miR-33	0.6342707	−0.3112329	0.9637398
hsa-miR-96	0.040926	0.5554684	
hsa-miR-28	1.1396825	1.0107414	
hsa-miR-30e-5p	0.3379079		
hsa-miR-199a*	−0.8762626		
hsa-miR-501	0.0812317	−0.167766	−0.6013733
hsa-miR-504	0.2946595	−0.1356475	−0.3858902
hsa-miR-15b	−0.1307195		
hsa-miR-29c	−0.1678	0.3820155	0.1279756
hsa-miR-455	−0.2162923	−0.2365768	
hsa-miR-380-3p	−0.1295169	−0.2513642	−0.2837673
hsa-miR-323	0.0956179	−0.3292096	
hsa-miR-527	0.3199353	−0.3980191	−0.4156484
hsa-miR-93	0.8926459	0.8342627	1.4247242
hsa-miR-32	−0.3081563		
hsa-miR-20b	−0.3025591		
hsa-miR-516-5p	0.1006308	−0.2462411	−0.4384718
hsa-miR-92	1.5916982		1.9080176
hsa-miR-30a-3p	0.2072069		
hsa-miR-497	0.1045716	−0.2486034	0.036478
hsa-miR-219	0.3630727	−0.2988121	
hsa-miR-499	−0.0609767	−0.3046793	−0.2561058
hsa-miR-149	0.654253	−0.2538624	0.04254
hsa-miR-30e-3p	0.4068861	−0.0672197	0.11598
hsa-miR-148a			
hsa-miR-520a	−0.0963095	−1.5626633	
hsa-miR-526b*	0.3595811	0.594503	

**Table 7:** Top-ranked miRNAs from Table 2, adjusted for covariate effects of medications (classes A, B, C, and B+C).

miRNA	SZ score (z-score from SAM)	p-value	Class A covariate adjust	p-value	Class B covariate adjust	p-value	Class C covariate adjust	p-value	Classes B+C covariate adjust	p-value
hsa-miR-31	3.55283	**0.0007**	4.10031	**0.00009**	3.55010	0.00073	3.55283	0.00072	3.5501	**0.0007**
hsa-miR-33	3.10046	**0.0032**	2.46619	**0.01906**	3.41170	0.00118	2.13672	0.04069	2.4480	**0.0199**
hsa-miR-96	2.98240	**0.0046**	2.94147	**0.00527**	2.42693	0.02099	2.98240	0.00467	2.4269	**0.0210**
hsa-miR-28	2.31611	**0.0272**	1.17643	0.19970	1.30537	0.17017	2.31611	0.02729	1.3054	0.1702
hsa-miR-30e-5p	2.37744	**0.0236**	2.03954	**0.04985**	2.37744	0.02363	2.37744	0.02363	2.3774	**0.0236**
hsa-miR-199a*	2.21841	**0.0340**	3.09467	**0.00332**	2.21841	0.03406	2.21841	0.03406	2.2184	**0.0341**
hsa-miR-501	−1.38651	0.1525	−1.46774	0.13587	−1.21874	0.18983	−0.78514	0.29312	−0.6174	0.3297
hsa-miR-504	−1.26857	0.1784	−1.56323	0.11756	−1.13292	0.20999	−0.88268	0.27022	−0.7470	0.3018
hsa-miR-15b	1.94092	0.0606	2.07164	**0.04666**	1.94092	0.06066	1.94092	0.06066	1.9409	0.0607
hsa-miR-29c	−1.25599	0.1812	−1.08819	0.22068	−1.63801	0.10430	−1.38397	0.15311	−1.7660	0.0839
hsa-miR-455	1.93342	0.0615	2.14972	**0.03957**	2.17000	0.03788	1.93342	0.06154	2.1700	**0.0379**
hsa-miR-380-3p	−0.78517	0.2931	−0.65565	0.32178	−0.53381	0.34597	−0.50140	0.35182	−0.2500	0.3867
hsa-miR-323	1.79862	0.0791	1.70300	0.09357	2.12783	0.04147	1.79862	0.07915	2.1278	**0.0415**
hsa-miR-527	−1.15943	0.2037	−1.47937	0.13356	−0.76141	0.29855	−0.74378	0.30254	−0.3458	0.3758
hsa-miR-93	1.59984	0.1109	0.70719	0.31068	0.76558	0.29760	0.17512	0.39287	−0.6591	0.3210
hsa-miR-32	1.70564	0.09315	2.01380	**0.05252**	1.70564	0.09315	1.70564	0.09315	1.7056	0.0931
hsa-miR-20b	1.87742	0.06847	2.17998	**0.03706**	1.87742	0.06847	1.87742	0.06847	1.8774	0.0685
hsa-miR-516-5p	−0.84614	0.27890	−0.94677	0.25484	−0.59990	0.33325	−0.40767	0.36713	−0.1614	0.3938
hsa-miR-92	1.56396	0.11743	−0.02774	0.39879	1.56396	0.11743	−0.34406	0.37601	−0.3441	0.3760
hsa-miR-30a-3p	1.67931	0.09740	1.47210	0.13500	1.67931	0.09740	1.67931	0.09740	1.6793	0.0974
hsa-miR-497	−0.56728	0.33965	−0.67185	0.31834	−0.31867	0.37919	−0.60376	0.33247	−0.3551	0.3746

The significance of results for miRNAs 31, 33, 96, 30e-5p, and 199a* (bold) was preserved (see Table 2), while miRNAs 15b, 455, 32, and 20b acquire significance (bold) after the adjustment for the drug class A (antipsychotics used in SZ only). After the adjustment for drug classes B (neurotransmitters receptor-site binders) and C (sedatives, hypnotics, anticonvulsants and analgesics) drug classes, the significance for miRNAs 31, 33, 96, 30e-5p, and 199a* (bold) was again preserved (see Table 2) while miR-455 acquired significance together with miR-323 (bold).

SZ =  schizophrenia.

**Table 8:** Top-ranked miRNAs from Table 3, adjusted for covariate effects of medications (classes B, C, and B+C).

miRNA	BD score (z-score from SAM)	p-value	Class B covariate adjust	p-value	Class C covariate adjust	p-value	Classes B+C covariate adjust	p-value
hsa-miR-219	−2.8237	**0.0074**	−2.8237	0.0074	−3.1225	0.0030	−3.1225	**0.0030**
hsa-miR-380-3p	−2.2933	**0.0287**	−2.0419	0.0496	−2.0095	0.0529	−1.7581	0.0850
hsa-miR-499	−2.2226	**0.0337**	−1.9179	0.0634	−1.9665	0.0576	−1.6618	0.1002
hsa-miR-497	−2.1947	**0.0358**	−1.9460	0.0600	−2.2311	0.0331	−1.9825	0.0558
hsa-miR-149	−2.1785	**0.0371**	−1.924	0.0625	−2.2211	0.0338	−1.9672	0.0576
hsa-miR-501	−2.0751	**0.0463**	−1.9073	0.0647	−1.4737	0.1346	−1.3059	0.1700
hsa-miR-29c	−2.0161	**0.0522**	−2.3981	0.0224	−2.1440	0.0400	−2.5260	**0.0164**
hsa-miR-30e-3p	−1.9787	0.0563	−2.0274	0.0444	−2.0947	−1.9115	0.0641	**0.0510**
hsa-miR-504	−1.9404	0.0607	−1.4189	0.1191	−1.5545	−1.8048	0.0782	0.1457
hsa-miR-148a	−1.8790	0.0682	−1.8790	0.0682	−1.8790	−1.8790	0.0682	0.0682
hsa-miR-520a	−1.8457	0.0726	−0.2830	0.0726	−1.8457	−0.2830	0.3832	0.3832
hsa-miR-526b*	−1.7745	0.0826	−2.3691	0.0826	−1.7745	−2.3691	0.0241	**0.0241**

The consideration of combined effects of B and C drug classes heightened the significance of the results for miRNAs 219 and 29c (bold; see Table 3), while miRNA 30e-3p and 526b* (bold) acquired significance.

BD =  bipolar disorder.

When adjusted for drug class A (antipsychotics used in SZ only), the significance of results for miRNAs 31, 33, 96, 30e-5p, and 199a* was preserved, while miRNAs 15b, 455, 32, and 20b acquire significance (Table 7, see also Table 2). After the adjustment for B (neurotransmitters receptor-site binders) and C (sedatives, hypnotics, anticonvulsants and analgesics) drug classes in SZ subjects, the significance for miRNAs 31, 33, 96, 30e-5p, and 199a* was still preserved while miR-455 (again) acquired significance together with miR-323 (Table 7, see also Table 2). In BD subjects, the consideration of combined effects of B and C drug classes heightened the significance of the results for miRNAs 219 and 29c while miRNA 30e-3p and 526b* acquired significance (Table 8, see also Table 3). Importantly, Wilcoxon test also indicated the expression of miRNAs 219 and 526b to be significantly different in BD cases in comparison to controls, but failed to discover miRNA 29c (Table S3; see Discussion).

Finally, while effects related to gender were not relevant to this study due to a male dominated cohort, we did consider the ages of our cases (Table 1). Although the SZ group of cases is on average older than BD and control groups, no significant difference in age between groups exists (Table S5).

### qPCR Analysis Confirms Differential Expression of miR-497 in SZ, and miR-29c in BD Samples in Comparison to Controls

**Figure 5:**
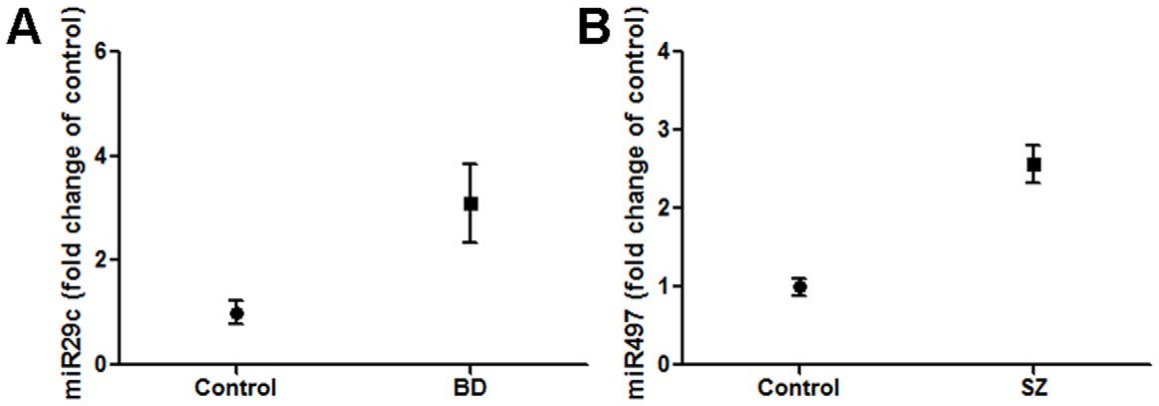
In comparison to controls, the expression of miR-29c and miR-497 is significantly increased in BD and SZ samples, respectively. Average exosomal RNA extracted from BA9 cortices of BD samples show a 2.77 fold increase of miR-29c in comparison to controls (A). SZ samples reveal 2.35 fold increase of miR-497 when compared to controls (B). Error bars indicate S.E.M.

To verify microarray data for miRNAs that may be differentially expressed in our three analyzed groups, controls, BD, and SZ, we performed a series of qPCR experiments. The primers for the following miRNAs worked in reactions with cDNA derived from each of our samples available for analysis: miR-31, -15b, -29c, -497, -219, and -149. These miRNAs were among the highest ranked according to the likelihood to be differentially expressed in both SZ and BD (miR-31, -15b, -29c, and -497; Table 2), or just BD (miR-219 and -149; Table 3). To further test the reproducibility of our data we used additional BD and additional control cases that became available through brain banks and BMC autopsy service (Table 1): SZ 1–6 (same as in *Luminex* experiment), BD 5–9 (three new samples), and C 6–13 (seven new samples). As shown in Table 9 and Figure 5, qPCR analyses confirmed miR-29c to be significantly differentially expressed (2.77 fold increased) in the examined BD samples, and miR-497 in SZ samples (2.35 fold increased) in comparison to controls.

**Table 9:** Student’s t-test comparison of qPCR Delta-CT.

miRNA	p-value, BD	Significant fold change	p-value, SZ	Significant fold change
hsa-miR-15b	0.6971		0.7906	
hsa-miR-29c	0.0237	2.77	0.501	
hsa-miR-31	0.426		0.3699	
hsa-miR-149	0.591		0.5011	
hsa-miR-219	0.8723		0.4059	
hsa-miR-497	0.1969		0.0026	2.35

miR-29c expression is significantly increased in BD samples, and miR-497 in SZ samples.

BD =  bipolar disorder; SZ =  schizophrenia.

## Discussion

While genetic component has been extensively researched in BD and SZ patients, the biological markers for these diseases have proven remarkably elusive. The putative susceptibility genes in both BD and SZ regulate cell-signaling [47]–[48]. This study analyzed the expression of miRNAs found in exosomes (Figure 1A and B), signaling vesicles capable of cell-to-cell transfer of miRNAs, in the prefrontal cortices of patients diagnosed with either BD or SZ and of individuals with no mental illnesses. Standard statistical analysis (Student’s t-test with Bonferroni Step Down/Holm correction) of the results of *Luminex* oligonucleotide- based screen for the expression of 312 miRNAs revealed only three miRNAs differentially expressed in SZ in comparison to BD and control samples (Table S1). While these three miRNAs (miR-31, -33, and -96) were also most highly ranked in a z-score based SAM, an additional 18 miRNAs were suggested to have truly differential expression (Table 2). Accordingly, the expression values of about 21 miRNAs were not false discoveries in a three-way comparison (Figure 2). These 21 miRNAs distinguished SZ but not BD samples from controls relatively well (Figure 3). Upon elimination of the distinctive influence of SZ samples in the three-way comparison (Table 2), the short list of most differentially expressed miRNA in BD compared to controls (Table 3) still featured several of the original top 21-ranked miRNAs. The covariate adjustment for the medications used by some of the subject cases (Tables 4 and 5), had nominal effects on significance of differential miRNA expression in the SZ group, while in the BD group the difference in the expression of miR-30e-3p, -219 and -29c attained heightened significance (Tables 6–8). To verify the microarray data, we performed qPCR analysis of the selected miRNAs in a partially new set of samples. Two miRNAs were confirmed to have significantly altered expression: in comparison to controls, the expression of miR-497 and miR-29c were significantly higher in SZ samples and BD samples, respectively (Table 9).

Our study has several novel and important findings. While exosomal content has been intensively studied in human diseases ever since the report of Valadi et al. in 2007 [30], we demonstrated here for the first time that exosomal miRNAs could be obtained from frozen postmortem brain tissue. On electron microscopy our PFC- derived exosome-containing pellets showed membrane bound vesicles within the range of the reported size of exosomes [49] (Figure 1A and B). In addition to the identification by size, shape, and miRNA content (intracellular micro-vesicles of similar size, e.g. lysosomes, do not contain miRNAs), we demonstrated a robust signal for CD63 and GAPDH in the micro-vesicles extracted from the human PFC (Figure 1A and B). According to Mathivanan and Simpson (2009) [50] exosomes from different sources have varying sets of protein markers on their surface, with CD63 being the second most, and GAPDH being the fourth most commonly reported exosomal antigen. Finally, our exosome-containing pellets were enriched with exosomal marker flotillin-2 [35] on Western blots, similar to the pellets from the medium of cultured cells (Figure 1C). Having insight into the cortical exosomal miRNA content may be of particular importance because exosomes are released into the CSF, and thus accessible for evaluation in living patients [51]. A recent study identified a substantial number of miRNAs exclusively or predominantly expressed in CSF [52]. Comparing the data from brain tissue with CSF will indeed require clinical investigation since postmortem brain depositories do not currently have sufficient number of samples and/or amount of CSF from neuropsychiatric patients. So far, the search for specifically altered miRNA expression in peripheral blood mononuclear cells (PBMCs) in SZ yielded diverse results [53], [54]. Three miRNAs were found to be differentially expressed in PBMCs as well as in PFC (BA46) of SZ patients [53]–[55].

The second novel and important feature of our investigation lies in the fact that our statistical analyses pointed to both differences and similarities between the miRNA expression changes in SZ and BD samples in comparison to control samples. The SAM top 21- ranked miRNAs (Table 2, Figure 2) had the ability to separate SZ samples, but not BD samples from the controls (Figure 3). This finding is directly connected to the suggested inherent differences in the variability of miRNA expression values in control samples in the three analyzed groups of samples, illustrated by the misclassification error rate analysis (Figure 4). The high variability of miRNA expression values in control samples represents perhaps a defining feature - the agility of the cortical transcriptional regulation in the absence of mental disease.

Initial statistical analysis revealed three miRNAs (miR-31, -33, and -96) differentially regulated in SZ samples (Table S1). Of those three, miR-33 was previously reported among misexpressed miRNAs in PFCs of both SZ and BD individuals [10]. Since we performed qPCR for additional miRNAs that were likely to be differentially expressed in SZ and BD samples according to SAM (Tables 2 and 3), we found significantly increased miR-497 expression in exosome-containing pellets from PFCs of SZ patients (Table 9). Perkins et al. (2007) [11] reported 16 miRNAs to be differentially expressed in PFCs of SZ subjects, one being miR-195. Both miR-195 and -497 belong to the well-studied miR-15/107 gene family. miR-15/107 miRNAs share most of their targets and are implicated in the pathogenesis of neoplasms, neurodegenerative diseases and heart disease [56]. Importantly, the up-regulation of these miRNAs expected to affect cortical gene expressions, has been reported in SZ [57]. Interestingly, miR-497 was also found to promote ischemic neuronal death by negatively regulating anti-apoptotic proteins, bcl-2 and bcl-w [58]. Three more miRNAs from our SAM top 21-ranked miRNAs were on the list of significantly down-regulated miRNAs in SZ by Perkins and colleagues [11]: miR-92, -20b, and -30e. miR-92 and -20b belong to miR-17-92 cluster known to be a potent oncogene [59]. Potentially pertinent for SZ pathobiology, miR-92 has also been suggested to developmentally regulate neuronal K(+)Cl(−) co-transporter 2 (KCC2) that modulates effects of GABA [60]. Finally, miR-30e may regulate neuronal death as down-regulation of miR-30e expression promoted neuronal survival in long-lived calorie-restricted mice [61]. However, we were not able to validate differential expression of miRNA-30e in the exosome-containing samples of PFCs in SZ (data not shown). We found miR-29c to be significantly increased in the exosome-enriched preparations from PFC of individuals diagnosed with BD in comparison to controls according to SAM and qPCR analysis. Interestingly, the expression of miR-29c was not significantly different in BD cases compared to controls according to Wilcoxon test applied to our *Luminex* data (p-value 0.0649; Table S3). The fact that miR-29c was significantly differentially expressed in SAM (Tables 2 and 3), further confirmed by qPCR analysis in BD vs. controls (Table 9), suggests that FDR testing through the use of SAM might be a superior strategy for discovery of true positives.

Like miR-497, the miRNA confirmed to be up-regulated in SZ samples, miR-29c was among the top-ranked in both SAM analyses (Table 2 and 3). In other words, both of these miRNAs were considered highly differentially regulated in both diseases according to SAM. Moreover, miR-29c was also reported by Perkins et al. to be differentially expressed miRNAs in PFC of SZ patients [11]. Interestingly, miR-29 together with miR-31, the top-ranked differentially expressed miRNA by SAM but not by qPCR (Table S1, Table 8), was proposed to regulate several cell-adhesion machinery components that are affected in the pathogenesis of many diseases [62]. The up-regulation of miR-29c in the cortical exosomes of BD patients is particularly intriguing because miR-29c is induced by canonical Wnt signaling [63] that is antagonized by GSK-3, a know substrate of inhibition by lithium, a first line of therapy for BD [64].

The interpretation of our findings has the following limitations. First, the relationship between exosomal and cellular miRNAs is not understood: is the exosomal miRNA aberration a part of pathogenesis, or a corrective attempt? Second, age-associated common brain pathology, may complicate the interpretation of our results, although vascular and early Alzheimer’s disease-associated pathologies were relatively evenly distributed in our samples (Table 1). Third, we were limited by a finite number of the samples available that fulfilled the RNA quality standards. However, the statistical methods we applied were developed specifically as a “non-parametric” test for miRNA expression studies. This test has a built-in mechanism for evaluating small sample sizes, and makes use of a “positive constant” standard error value as one of its formulaic variables. This ensures that miRNAs with small values are not elevated high in the rankings simply because they have a small standard error. By comparison t-tests often assign strong significance to miRNAs with small expression levels due to their small standard errors. The PAM and SAM programs we used are therefore optimal in dealing with irregular data with small sample size such as ours.

Fourth, our microarray examined the expression of 312 miRNA, while hundreds more are known to exist and thus may be important for the pathobiology of SZ and BD. Finally, due to the variable efficiency of the primers in our cDNA samples, we could carry qPCRs for only six miRNAs (Table 8, 9) featured among our top-ranked in SAM analyses (Tables 2 and 3). Nevertheless, the results of qPCR validation are remarkable considering that we examined different samples from the ones analyzed in the microarray, in particular controls - most likely to exhibit highly variable miRNA expressions (Figure 4). Interestingly, our qPCR analysis did not validate SZ-associated highly significant up-regulation of miR-31 by Luminex assay (Table 2, Table S1). Considering, however, that Gardiner et al. (2012) [54] confirmed miR-31 down-regulation in PBMCs of SZ patients, miR-31 dysregulation in SZ cannot be ruled-out. Our search for exosome-derived biomarkers yielded two candidates, miR-497 and miR-29c, in the PFC of SZ and BD patients respectively, potentially offering a novel insight into the pathogenesis of these diseases. In addition, rapid development of exosome nanotechnology [65] adds the promise of alternative therapeutics delivery [66]–[67] to the emerging diagnostic potential of brain-derived exosomes in neuropsychiatric diseases.

## Acknowledgments

We thank George Tejada and David Ennulat of Harvard Brain Tissue Resource Center and Michiel Kooreman of The Netherlands Brain Bank for tissue and clinical histories procurement, Maria Ericsson and Howard Mulhern for expert electron microscopy processing, Ozge Cagsal-Getkin for help with *Luminex* assay, and Zhigang Xie for critical reading of the manuscript.

## Supporting Information

Figure S1 **RNA quality control.** Representative RNA (sample C13, RIN = 7.0; the sample closest to the average (6.96) and median (6.85) value for the set) yields two strong bands at 40 s and 45 s representing the 18 S and 28 S ribosomal RNA (A). The electropherogram shows a marker peak and the two ribosomal peaks corresponding to 18 S and 28 S subunits. RIN value on a scale of 1–10 is computed based on the presence of correct ribosomal peaks, the ratio between those peaks, and the extent of RNA fragmentation (B). Relationship between RIN and mRNA and miRNA profiles (C): Next to the ladder (left), total and small RNA profiles of three degradation stages of a single RNA sample are shown (RIN 8.4 =  not degraded, RIN 3.1 =  partially degraded, RIN 2.5 =  severely degraded). Note that both mRNA (RT PCR, middle) and miRNA (*Luminex,* right) profiles obtained from a sample with RIN 3.1 (low in comparison to the average RIN of 6.96 in this study) are still relatively similar to the profile obtained from a sample with high (8.4) RIN.
(TIF)

Figure S2 **RNase treatment effect on total RNA profile.** *Agilent RNA 6000 Pico* chips have the ability to resolve RNAs in the size range of 25 nt to 6000 nt (top row). *Agilent Small RNA* chips have superior resolving power for RNAs in the range of 4 nt to 150 nt only (bottom row). Electropherogram from *Pico* chip shows that in addition to miRNAs, our exosome preparations also contain higher molecular weight cellular RNAs of sizes up to 4000 nt. These higher molecular weight species cannot be seen with the small RNA chips. Digesting exosome preparations with RNase A or a combination of RNase A and RNase T1 destroys the higher molecular weight extra-exosomal cellular RNAs and preserves only the small RNAs contained in the exosome itself. Note, also, that the fluorescent units scale [FU] on the Y-axis of all of the electropherograms represents the quantity of RNA. As such, RNase treated preps contain much less RNA than untreated preps as the exosomal RNA represents a small portion of the cellular RNA. Similarly, RNase digestion reduces both the size and amount of small RNAs present in exosomal preparations. Exosomal RNA preparations not treated with RNase contain more and larger RNAs in the 4–150+ nt range than do preparations treated with RNase A alone or RNases A and T1. The expected size ranges of miRNAs (∼19–28 nt, yellow) and pre-miRNAs (45–60 nt, blue) are indicated in the small RNA assay panels (bottom row). RNAs in these size ranges are well represented in our purified exosomal RNA preparations.
(TIF)

Figure S3 ***NCode*** **amplification of exosome-derived RNA.** Amplification of miRNAs (which have no poly-A tails) requires the addition of a 3′ Oligo(dT) 24-mer as well as a 5′ T7 promoter template, which increases the size of each miRNA by ∼40 nt. In this case, the expected size range of amplified miRNAs is ∼60 nt (arrow) as opposed to that of the native ∼20 nt species. Profiles of amplified miRNA upon treatment with RNase A (green), without RNase (blue), and with RNase A/T1 (red – optimal for exosome-derived RNA used in *Luminex* assay) are similar.
(TIF)

Table S1 **Student’s T tests of** ***Luminex*** **miRNA expression data with corrected p-values.**
(DOCX)

Table S2 **Quantities of exosome-derived miRNA from the analyzed cases for** ***Luminex*** **assay, before and after** ***NCode*** **amplification.**
(DOCX)

Table S3 **Wilcoxon non-parametric test and fold change for top BD hits as means for statistical verification.** 12 top-ranked miRNAs according to SAM in Table 3, with q-values lower than 15%, ending with miR-526b* - the last miRNA to have significantly changed expression in BD according to this test. miRNAs 219, 380–3p, 148a, 520a, and 526b* (p-values in bold) have significantly changed expression in BD cases in comparison to controls by this analysis. The expression of miR-29c is not significantly different in BD cases according to Wilcoxon test (p-value 0.0649; see Discussion).
(DOCX)

Table S4 **Phenotype traits (tobacco, alcohol, and recreational drug use) of analyzed cases.**
(DOCX)

Table S5 **An ANOVA analysis of differences in age between C, BD, and SZ groups of cases.** No significant difference in age between the groups exists.
(DOCX)
