# Supplementary figures and images for: Differential Expression of Exosomal microRNAs in Prefrontal Cortices of Schizophrenia and Bipolar Disorder Patients
Source: PLoS One. 2013 Jan 30;8(1):e48814. doi: 10.1371/journal.pone.0048814 (PMC3559697; doi:10.1371/journal.pone.0048814)

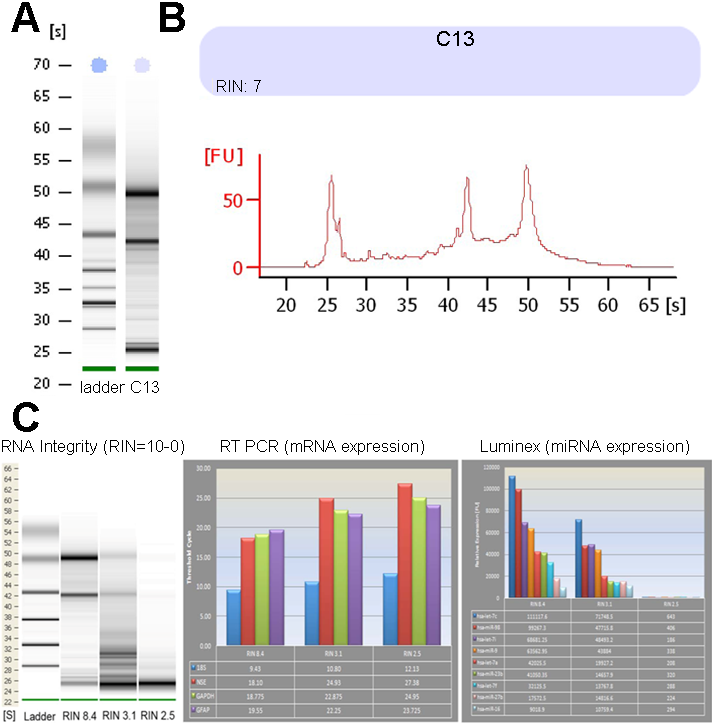

Supplement: Figure S1 — RNA quality control. Representative RNA (sample C13, RIN = 7.0; the sample closest to the average (6.96) and median (6.85) value for the set) yields two strong bands at 40 s and 45 s representing the 18 S and 28 S ribosomal RNA (A). The electropherogram shows a marker peak and the two ribosomal peaks corresponding to 18 S and 28 S subunits. RIN value on a scale of 1–10 is computed based on the presence of correct ribosomal peaks, the ratio between those peaks, and the extent of RNA fragmentation (B). Relationship between RIN and mRNA and miRNA profiles (C): Next to the ladder (left), total and small RNA profiles of three degradation stages of a single RNA sample are shown (RIN 8.4 = not degraded, RIN 3.1 = partially degraded, RIN 2.5 = severely degraded). Note that both mRNA (RT PCR, middle) and miRNA (Luminex, right) profiles obtained from a sample with RIN 3.1 (low in comparison to the average RIN of 6.96 in this study) are still relatively similar to the profile obtained from a sample with high (8.4) RIN. (TIF) [file pone.0048814.s001.tif]

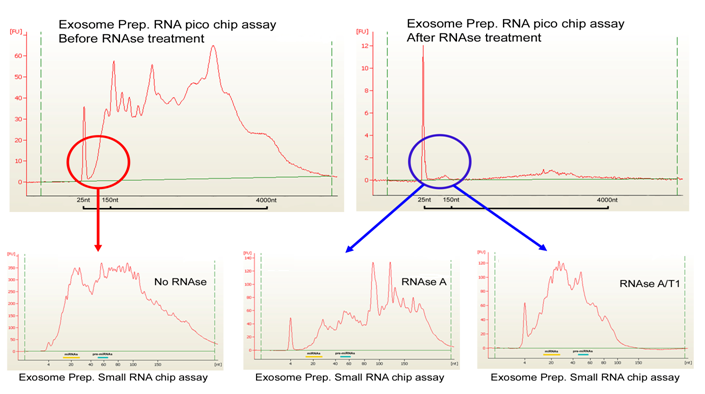

Supplement: Figure S2 — RNase treatment effect on total RNA profile. Agilent RNA 6000 Pico chips have the ability to resolve RNAs in the size range of 25 nt to 6000 nt (top row). Agilent Small RNA chips have superior resolving power for RNAs in the range of 4 nt to 150 nt only (bottom row). Electropherogram from Pico chip shows that in addition to miRNAs, our exosome preparations also contain higher molecular weight cellular RNAs of sizes up to 4000 nt. These higher molecular weight species cannot be seen with the small RNA chips. Digesting exosome preparations with RNase A or a combination of RNase A and RNase T1 destroys the higher molecular weight extra-exosomal cellular RNAs and preserves only the small RNAs contained in the exosome itself. Note, also, that the fluorescent units scale [FU] on the Y-axis of all of the electropherograms represents the quantity of RNA. As such, RNase treated preps contain much less RNA than untreated preps as the exosomal RNA represents a small portion of the cellular RNA. Similarly, RNase digestion reduces both the size and amount of small RNAs present in exosomal preparations. Exosomal RNA preparations not treated with RNase contain more and larger RNAs in the 4–150+ nt range than do preparations treated with RNase A alone or RNases A and T1. The expected size ranges of miRNAs (∼19–28 nt, yellow) and pre-miRNAs (45–60 nt, blue) are indicated in the small RNA assay panels (bottom row). RNAs in these size ranges are well represented in our purified exosomal RNA preparations. (TIF) [file pone.0048814.s002.tif]

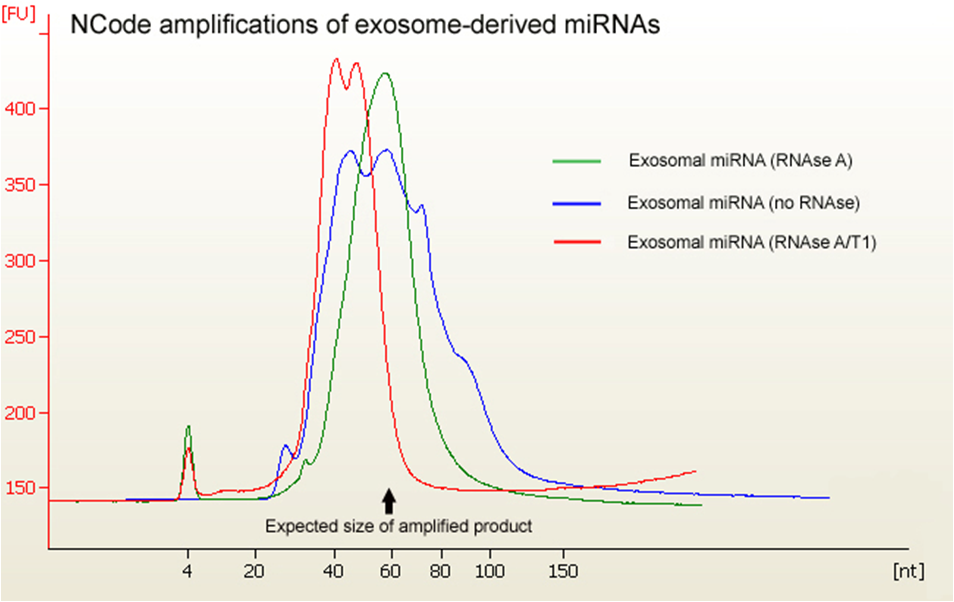

Supplement: Figure S3 — NCode amplification of exosome-derived RNA. Amplification of miRNAs (which have no poly-A tails) requires the addition of a 3′ Oligo(dT) 24-mer as well as a 5′ T7 promoter template, which increases the size of each miRNA by ∼40 nt. In this case, the expected size range of amplified miRNAs is ∼60 nt (arrow) as opposed to that of the native ∼20 nt species. Profiles of amplified miRNA upon treatment with RNase A (green), without RNase (blue), and with RNase A/T1 (red – optimal for exosome-derived RNA used in Luminex assay) are similar. (TIF) [file pone.0048814.s003.tif]
