# Supplementary material for: Differential Expression of Exosomal microRNAs in Prefrontal Cortices of Schizophrenia and Bipolar Disorder Patients
Source: PLoS One. 2013 Jan 30;8(1):e48814. doi: 10.1371/journal.pone.0048814 (PMC3559697; doi:10.1371/journal.pone.0048814)
Supplement: Table S1 — Student’s T tests of Luminex miRNA expression data with corrected p-values. (DOCX) [file pone.0048814.s004.docx]

|  | C |  |  | BD |  |  |  |  | New threshold/Significance | SZ |  |  |  |  | New threshold/Significance |
| --- | --- | --- | --- | --- | --- | --- | --- | --- | --- | --- | --- | --- | --- | --- | --- |
| miRNA | n | Avg | Variance | n | Avg | Variance | t [C:BD] | df=10 | **Bonferroni Step Down (Holm) Correction** | n | Avg | Variance | t [C:SZ] | df=12 | **Bonferroni Step Down (Holm) Correction** |
|  |  |  |  |  |  |  |  | a=.10 |  |  |  |  |  | a=.10 |  |
| hsa-let-7a | 6 | 164 | 32268 | 6 | 409 | 204386 | -1.232 |  |  | 8 | 350 | 62027 | -1.625 |  |  |
| hsa-let-7b | 6 | 708 | 759694 | 6 | 585 | 201410 | 0.307 |  |  | 8 | 732 | 173474 | -0.063 |  |  |
| hsa-let-7c | 6 | 429 | 263338 | 6 | 670 | 266334 | -0.813 |  |  | 8 | 467 | 127236 | -0.157 |  |  |
| hsa-let-7d | 6 | 927 | 1137923 | 6 | 1077 | 744900 | -0.268 |  |  | 8 | 1133 | 339089 | -0.428 |  |  |
| hsa-let-7e | 6 | 2704 | 9057495 | 6 | 1887 | 579501 | 0.644 |  |  | 8 | 2115 | 2051150 | 0.443 |  |  |
| hsa-let-7f | 6 | 75 | 14384 | 6 | 192 | 77640 | -0.941 |  |  | 8 | 297 | 27435 | -2.913 | ** | 0.000180505/ NO |
| hsa-let-7g | 6 | 93 | 2727 | 6 | 141 | 7363 | -1.181 |  |  | 8 | 167 | 22975 | -1.290 |  |  |
| hsa-let-7i | 6 | 70 | 17728 | 6 | 108 | 11437 | -0.539 |  |  | 8 | 176 | 30404 | -1.285 |  |  |
| hsa-miR-1 | 6 | 304 | 62796 | 6 | 281 | 48649 | 0.168 |  |  | 8 | 284 | 25098 | 0.166 |  |  |
| hsa-miR-100 | 6 | 133 | 8527 | 6 | 63 | 2234 | 1.663 |  |  | 8 | 178 | 8762 | -0.896 |  |  |
| hsa-miR-103 | 6 | 317 | 101883 | 6 | 275 | 63023 | 0.252 |  |  | 8 | 557 | 36406 | -1.637 |  |  |
| hsa-miR-105 | 6 | 127 | 4586 | 6 | 98 | 14376 | 0.517 |  |  | 8 | 8 | 78 | 4.301 | *** | 0.000164474/ NO |
| hsa-miR-10a | 6 | 149 | 2961 | 6 | 158 | 8014 | -0.224 |  |  | 8 | 277 | 29920 | -1.967 | * | 0.000212766/ NO |
| hsa-miR-10b | 6 | 65 | 6395 | 6 | 34 | 1187 | 0.867 |  |  | 8 | 216 | 20634 | -2.496 | ** | 0.000189394/ NO |
| hsa-miR-133a | 6 | 265 | 81803 | 6 | 258 | 64972 | 0.050 |  |  | 8 | 186 | 32368 | 0.599 |  |  |
| hsa-miR-133b | 6 | 24 | 2711 | 6 | 56 | 1910 | -1.156 |  |  | 8 | 176 | 11411 | -3.522 | *** | 0.000170648/ NO |
| hsa-miR-142-3p | 6 | 30 | 982 | 6 | 5 | 63 | 1.926 |  |  | 8 | 63 | 3308 | -1.384 |  |  |
| hsa-miR-15a | 6 | 188 | 3915 | 6 | 191 | 6023 | -0.072 |  |  | 8 | 229 | 14764 | -0.816 |  |  |
| hsa-miR-15b | 6 | 49 | 1920 | 6 | 0 | 0 | 2.758 | ** | 0.000160772/ NO | 8 | 198 | 13798 | -3.282 | *** | 0.000172414/ NO |
| hsa-miR-16 | 6 | 104 | 5774 | 6 | 128 | 3077 | -0.638 |  |  | 8 | 222 | 12382 | -2.363 | ** | 0.000192308/ NO |
| hsa-miR-184 | 6 | 2818 | 8769256 | 6 | 2465 | 1591692 | 0.269 |  |  | 8 | 1952 | 1696236 | 0.670 |  |  |
| hsa-miR-18a | 6 | 113 | 1124 | 6 | 88 | 7138 | 0.687 |  |  | 8 | 140 | 6204 | -0.872 |  |  |
| hsa-miR-18a* | 6 | 32 | 5024 | 6 | 45 | 6222 | -0.294 |  |  | 8 | 144 | 7728 | -2.647 | ** | 0.000183824/ NO |
| hsa-miR-18b | 6 | 109 | 20049 | 6 | 48 | 3238 | 0.976 |  |  | 8 | 126 | 11322 | -0.243 |  |  |
| hsa-miR-199a | 6 | 354 | 6051 | 6 | 431 | 41260 | -0.861 |  |  | 8 | 308 | 22432 | 0.747 |  |  |
| hsa-miR-199b | 6 | 106 | 2469 | 6 | 102 | 7471 | 0.080 |  |  | 8 | 267 | 20919 | -2.928 | ** | 0.000179211/ NO |
| hsa-miR-20a | 6 | 106 | 5001 | 6 | 73 | 3596 | 0.892 |  |  | 8 | 145 | 17728 | -0.699 |  |  |
| hsa-miR-20b | 6 | 42 | 1858 | 6 | 41 | 1144 | 0.011 |  |  | 8 | 173 | 7249 | -3.775 | *** | 0.00016835/ NO |
| hsa-miR-214 | 6 | 379 | 251386 | 6 | 663 | 429940 | -0.844 |  |  | 8 | 635 | 121527 | -1.073 |  |  |
| hsa-miR-23a | 6 | 456 | 530454 | 6 | 293 | 30086 | 0.532 |  |  | 8 | 365 | 28996 | 0.298 |  |  |
| hsa-miR-23b | 6 | 325 | 261714 | 6 | 143 | 29312 | 0.829 |  |  | 8 | 248 | 26656 | 0.355 |  |  |
| hsa-miR-25 | 6 | 0 | 0 | 6 | 2 | 17 | -0.949 |  |  | 8 | 85 | 13999 | -2.020 | * | 0.000207469/ NO |
| hsa-miR-27a | 6 | 1 | 2 | 6 | 23 | 3151 | -0.978 |  |  | 8 | 55 | 7149 | -1.812 | * | 0.00021645/ NO |
| hsa-miR-27b | 6 | 258 | 10041 | 6 | 289 | 8592 | -0.570 |  |  | 8 | 256 | 11824 | 0.035 |  |  |
| hsa-miR-28 | 6 | 66 | 7412 | 6 | 45 | 4870 | 0.464 |  |  | 8 | 352 | 22423 | -4.496 | *** | 0.000162866/ NO |
| hsa-miR-302b* | 6 | 690 | 528352 | 6 | 386 | 115797 | 0.928 |  |  | 8 | 711 | 140393 | -0.064 |  |  |
| hsa-miR-30a-3p | 6 | 10 | 401 | 6 | 18 | 796 | -0.543 |  |  | 8 | 167 | 14902 | -3.562 | *** | 0.000170068/ NO |
| hsa-miR-30e-5p | 6 | 90 | 565 | 6 | 41 | 1966 | 2.374 | ** | 0.000163399/ NO | 8 | 234 | 7833 | -4.415 | *** | 0.000163934/ NO |
| hsa-miR-31 | 6 | 38 | 3005 | 6 | 12 | 888 | 1.017 |  |  | 8 | 473 | 27482 | -6.926 | *** | 0.000160256/ YES |
| hsa-miR-32 | 6 | 14 | 1107 | 6 | 0 | 0 | 1.000 |  |  | 8 | 163 | 15247 | -3.272 | *** | 0.000173611/ NO |
| hsa-miR-323 | 6 | 31 | 419 | 6 | 52 | 2236 | -1.038 |  |  | 8 | 204 | 13774 | -4.109 | *** | 0.000165563/ NO |
| hsa-miR-33 | 6 | 0 | 0 | 6 | 10 | 313 | -1.316 |  |  | 8 | 229 | 9767 | -6.558 | *** | 0.000160772/ YES |
| hsa-miR-34a | 6 | 82 | 10445 | 6 | 104 | 11752 | -0.370 |  |  | 8 | 172 | 12945 | -1.555 |  |  |
| hsa-miR-34b | 6 | 33 | 1361 | 6 | 77 | 3663 | -1.506 |  |  | 8 | 43 | 3418 | -0.390 |  |  |
| hsa-miR-34c | 6 | 19 | 2185 | 6 | 31 | 1092 | -0.517 |  |  | 8 | 15 | 1172 | 0.183 |  |  |
| hsa-miR-431 | 6 | 3 | 29 | 6 | 0 | 0 | 1.209 |  |  | 8 | 65 | 4538 | -2.601 | ** | 0.000184502/ NO |
| hsa-miR-452* | 6 | 31 | 1709 | 6 | 10 | 247 | 1.186 |  |  | 8 | 71 | 4696 | -1.345 |  |  |
| hsa-miR-489 | 6 | 362 | 241737 | 6 | 516 | 341016 | -0.494 |  |  | 8 | 764 | 301218 | -1.439 |  |  |
| hsa-miR-517* | 6 | 244 | 86438 | 6 | 336 | 91022 | -0.532 |  |  | 8 | 469 | 55208 | -1.542 |  |  |
| hsa-miR-7 | 6 | 310 | 29923 | 6 | 241 | 22457 | 0.744 |  |  | 8 | 268 | 24705 | 0.465 |  |  |
| hsa-miR-9 | 6 | 278 | 9185 | 6 | 361 | 43346 | -0.881 |  |  | 8 | 266 | 15038 | 0.199 |  |  |
| hsa-miR-92 | 6 | 2 | 24 | 6 | 13 | 1014 | -0.836 |  |  | 8 | 190 | 25304 | -3.345 | *** | 0.000171821/ NO |
| hsa-miR-93 | 6 | 2 | 17 | 6 | 35 | 1515 | -2.081 | * | 0.00016835/ NO | 8 | 195 | 22244 | -3.669 | *** | 0.000168919/ NO |
| hsa-miR-95 | 6 | 211 | 2245 | 6 | 230 | 1473 | -0.780 |  |  | 8 | 234 | 8526 | -0.629 |  |  |
| hsa-miR-96 | 6 | 67 | 4321 | 6 | 68 | 3759 | -0.014 |  |  | 8 | 299 | 5745 | -6.117 | *** | 0.00016129/ YES |
| hsa-miR-98 | 6 | 489 | 443274 | 6 | 874 | 719705 | -0.877 |  |  | 8 | 894 | 292262 | -1.221 |  |  |
| hsa-miR-99b | 6 | 13 | 793 | 6 | 33 | 1517 | -1.024 |  |  | 8 | 12 | 683 | 0.025 |  |  |
| hsa-miR-107 | 6 | 48 | 3680 | 6 | 69 | 9245 | -0.444 |  |  | 8 | 65 | 2995 | -0.533 |  |  |
| hsa-miR-122a | 6 | 285 | 105065 | 6 | 422 | 329880 | -0.506 |  |  | 8 | 272 | 87784 | 0.079 |  |  |
| hsa-miR-126 | 6 | 97 | 8300 | 6 | 131 | 27665 | -0.442 |  |  | 8 | 58 | 2315 | 0.951 |  |  |
| hsa-miR-127 | 6 | 61 | 5861 | 6 | 94 | 18386 | -0.515 |  |  | 8 | 148 | 3655 | -2.281 | ** | 0.000195313/ NO |
| hsa-miR-128a | 6 | 85 | 7744 | 6 | 124 | 35803 | -0.458 |  |  | 8 | 90 | 1931 | -0.137 |  |  |
| hsa-miR-128b | 6 | 47 | 3962 | 6 | 74 | 16424 | -0.470 |  |  | 8 | 78 | 3357 | -0.951 |  |  |
| hsa-miR-129 | 6 | 30 | 717 | 6 | 39 | 1912 | -0.418 |  |  | 8 | 21 | 844 | 0.566 |  |  |
| hsa-miR-130a | 6 | 123 | 29008 | 6 | 196 | 85697 | -0.528 |  |  | 8 | 130 | 7340 | -0.091 |  |  |
| hsa-miR-130b | 6 | 26 | 1658 | 6 | 45 | 5550 | -0.535 |  |  | 8 | 12 | 233 | 0.806 |  |  |
| hsa-miR-132 | 6 | 53 | 3848 | 6 | 69 | 8154 | -0.355 |  |  | 8 | 20 | 416 | 1.263 |  |  |
| hsa-miR-134 | 6 | 354 | 200282 | 6 | 540 | 590501 | -0.513 |  |  | 8 | 195 | 21714 | 0.838 |  |  |
| hsa-miR-135b | 6 | 141 | 19996 | 6 | 196 | 60295 | -0.469 |  |  | 8 | 13 | 356 | 2.213 | ** | 0.0002/ NO |
| hsa-miR-136 | 6 | 19 | 889 | 6 | 20 | 1374 | -0.071 |  |  | 8 | 24 | 993 | -0.327 |  |  |
| hsa-miR-137 | 6 | 57 | 7464 | 6 | 98 | 28308 | -0.526 |  |  | 8 | 14 | 447 | 1.187 |  |  |
| hsa-miR-140 | 6 | 83 | 5723 | 6 | 112 | 17008 | -0.467 |  |  | 8 | 79 | 2432 | 0.128 |  |  |
| hsa-miR-141 | 6 | 62 | 10820 | 6 | 107 | 44646 | -0.467 |  |  | 8 | 130 | 5635 | -1.361 |  |  |
| hsa-miR-143 | 6 | 116 | 27151 | 6 | 184 | 78101 | -0.521 |  |  | 8 | 185 | 8432 | -0.929 |  |  |
| hsa-miR-145 | 6 | 14 | 476 | 6 | 18 | 1446 | -0.217 |  |  | 8 | 7 | 246 | 0.628 |  |  |
| hsa-miR-146a | 6 | 85 | 23860 | 6 | 124 | 46166 | -0.360 |  |  | 8 | 162 | 18824 | -0.973 |  |  |
| hsa-miR-146b | 6 | 83 | 7414 | 6 | 119 | 26460 | -0.488 |  |  | 8 | 88 | 3883 | -0.142 |  |  |
| hsa-miR-147 | 6 | 104 | 19944 | 6 | 159 | 52463 | -0.500 |  |  | 8 | 59 | 2550 | 0.744 |  |  |
| hsa-miR-150 | 6 | 350 | 194407 | 6 | 515 | 505976 | -0.483 |  |  | 8 | 186 | 24781 | 0.867 |  |  |
| hsa-miR-151 | 6 | 15 | 668 | 6 | 18 | 1169 | -0.163 |  |  | 8 | 8 | 173 | 0.628 |  |  |
| hsa-miR-152 | 6 | 25 | 3647 | 6 | 49 | 14152 | -0.439 |  |  | 8 | 14 | 638 | 0.420 |  |  |
| hsa-miR-153 | 6 | 18 | 169 | 6 | 15 | 238 | 0.356 |  |  | 8 | 69 | 1733 | -3.254 | *** | 0.000174216/ NO |
| hsa-miR-154 | 6 | 74 | 7971 | 6 | 115 | 33850 | -0.487 |  |  | 8 | 61 | 1969 | 0.332 |  |  |
| hsa-miR-154* | 6 | 52 | 6406 | 6 | 86 | 18664 | -0.535 |  |  | 8 | 45 | 1960 | 0.179 |  |  |
| hsa-miR-155 | 6 | 260 | 109239 | 6 | 409 | 385035 | -0.520 |  |  | 8 | 224 | 20974 | 0.245 |  |  |
| hsa-miR-182 | 6 | 17 | 1078 | 6 | 30 | 4406 | -0.415 |  |  | 8 | 102 | 2555 | -3.780 | *** | 0.000167785/ NO |
| hsa-miR-183 | 6 | 98 | 11440 | 6 | 145 | 46613 | -0.481 |  |  | 8 | 27 | 1124 | 1.572 |  |  |
| hsa-miR-185 | 6 | 1038 | 1579598 | 6 | 1536 | 4513037 | -0.494 |  |  | 8 | 592 | 131432 | 0.845 |  |  |
| hsa-miR-187 | 6 | 116 | 14409 | 6 | 165 | 48871 | -0.480 |  |  | 8 | 231 | 24215 | -1.560 |  |  |
| hsa-miR-188 | 6 | 39 | 5340 | 6 | 58 | 10298 | -0.373 |  |  | 8 | 91 | 6489 | -1.254 |  |  |
| hsa-miR-189 | 6 | 8 | 261 | 6 | 10 | 497 | -0.190 |  |  | 8 | 42 | 1152 | -2.507 | ** | 0.000187266/ NO |
| hsa-miR-190 | 6 | 8 | 165 | 6 | 11 | 405 | -0.268 |  |  | 8 | 87 | 2231 | -4.485 | *** | 0.000163399/ NO |
| hsa-miR-191 | 6 | 48 | 3265 | 6 | 72 | 10793 | -0.494 |  |  | 8 | 101 | 2339 | -1.846 | * | 0.000215517/ NO |
| hsa-miR-191* | 6 | 49 | 4178 | 6 | 75 | 15929 | -0.459 |  |  | 8 | 77 | 1508 | -0.933 |  |  |
| hsa-miR-192 | 6 | 204 | 34570 | 6 | 281 | 129109 | -0.470 |  |  | 8 | 206 | 11704 | -0.030 |  |  |
| hsa-miR-193a | 6 | 85 | 6941 | 6 | 120 | 25028 | -0.488 |  |  | 8 | 100 | 4118 | -0.379 |  |  |
| hsa-miR-193b | 6 | 32 | 3489 | 6 | 46 | 6670 | -0.327 |  |  | 8 | 87 | 3814 | -1.690 |  |  |
| hsa-miR-194 | 6 | 81 | 11593 | 6 | 116 | 26692 | -0.436 |  |  | 8 | 94 | 3092 | -0.283 |  |  |
| hsa-miR-195 | 6 | 120 | 19905 | 6 | 182 | 64480 | -0.520 |  |  | 8 | 103 | 3686 | 0.274 |  |  |
| hsa-miR-199a* | 6 | 11 | 142 | 6 | 15 | 566 | -0.376 |  |  | 8 | 194 | 12234 | -4.652 | *** | 0.000162338/ NO |
| hsa-miR-203 | 6 | 150 | 24736 | 6 | 214 | 78683 | -0.491 |  |  | 8 | 112 | 5409 | 0.543 |  |  |
| hsa-miR-208 | 6 | 138 | 28757 | 6 | 215 | 102835 | -0.523 |  |  | 8 | 177 | 11008 | -0.503 |  |  |
| hsa-miR-21 | 6 | 179 | 31640 | 6 | 255 | 110883 | -0.495 |  |  | 8 | 233 | 15332 | -0.635 |  |  |
| hsa-miR-220 | 6 | 56 | 5863 | 6 | 79 | 15051 | -0.380 |  |  | 8 | 82 | 2167 | -0.732 |  |  |
| hsa-miR-30a-5p | 6 | 84 | 10760 | 6 | 127 | 39809 | -0.468 |  |  | 8 | 57 | 1898 | 0.605 |  |  |
| hsa-miR-373* | 6 | 9384 | 161921649 | 6 | 14656 | 471201927 | -0.513 |  |  | 8 | 4177 | 36787837 | 0.927 |  |  |
| hsa-miR-378 | 6 | 64 | 6682 | 6 | 102 | 26560 | -0.499 |  |  | 8 | 64 | 990 | 0.012 |  |  |
| hsa-miR-410 | 6 | 39 | 1509 | 6 | 55 | 5461 | -0.458 |  |  | 8 | 75 | 3421 | -1.355 |  |  |
| hsa-miR-448 | 6 | 94 | 14926 | 6 | 150 | 61960 | -0.495 |  |  | 8 | 58 | 2820 | 0.669 |  |  |
| hsa-miR-452 | 6 | 972 | 1704689 | 6 | 1494 | 4702470 | -0.505 |  |  | 8 | 532 | 144752 | 0.802 |  |  |
| hsa-miR-485-5p | 6 | 65 | 4548 | 6 | 95 | 15536 | -0.511 |  |  | 8 | 52 | 1721 | 0.439 |  |  |
| hsa-miR-515-5p | 6 | 821 | 408176 | 6 | 1092 | 1805829 | -0.446 |  |  | 8 | 546 | 98747 | 0.973 |  |  |
| hsa-miR-518c* | 6 | 1212 | 2707671 | 6 | 1943 | 8963297 | -0.524 |  |  | 8 | 659 | 591913 | 0.763 |  |  |
| hsa-miR-518f* | 6 | 1164 | 2634503 | 6 | 1889 | 8694458 | -0.528 |  |  | 8 | 717 | 300143 | 0.647 |  |  |
| hsa-miR-519e* | 6 | 251 | 117125 | 6 | 397 | 347574 | -0.522 |  |  | 8 | 204 | 20298 | 0.320 |  |  |
| hsa-miR-524* | 6 | 356 | 218437 | 6 | 550 | 626138 | -0.517 |  |  | 8 | 212 | 16204 | 0.735 |  |  |
| hsa-miR-525 | 6 | 213 | 78862 | 6 | 335 | 245234 | -0.523 |  |  | 8 | 172 | 9407 | 0.343 |  |  |
| hsa-miR-526a | 6 | 457 | 391330 | 6 | 739 | 1329390 | -0.525 |  |  | 8 | 303 | 51845 | 0.579 |  |  |
| hsa-miR-526b | 6 | 630 | 816723 | 6 | 985 | 2146684 | -0.504 |  |  | 8 | 316 | 71128 | 0.826 |  |  |
| hsa-miR-526c | 6 | 1814 | 5040156 | 6 | 2727 | 14785224 | -0.502 |  |  | 8 | 1125 | 538006 | 0.723 |  |  |
| hsa-miR-138 | 6 | 74 | 8403 | 6 | 94 | 7192 | -0.382 |  |  | 8 | 125 | 6787 | -1.070 |  |  |
| hsa-miR-142-5p | 6 | 195 | 26937 | 6 | 281 | 48714 | -0.767 |  |  | 8 | 153 | 14499 | 0.527 |  |  |
| hsa-miR-186 | 6 | 285 | 29879 | 6 | 446 | 58750 | -1.325 |  |  | 8 | 286 | 38065 | -0.019 |  |  |
| hsa-miR-196a | 6 | 122 | 24625 | 6 | 128 | 11952 | -0.069 |  |  | 8 | 89 | 2837 | 0.502 |  |  |
| hsa-miR-196b | 6 | 24 | 316 | 6 | 91 | 9292 | -1.676 |  |  | 8 | 42 | 1966 | -1.081 |  |  |
| hsa-miR-197 | 6 | 111 | 10057 | 6 | 192 | 38552 | -0.902 |  |  | 8 | 183 | 14664 | -1.214 |  |  |
| hsa-miR-198 | 6 | 291 | 148664 | 6 | 463 | 191341 | -0.722 |  |  | 8 | 378 | 135476 | -0.424 |  |  |
| hsa-miR-200a* | 6 | 88 | 4507 | 6 | 97 | 17950 | -0.144 |  |  | 8 | 173 | 26012 | -1.349 |  |  |
| hsa-miR-202 | 6 | 6442 | 15207824 | 6 | 10663 | 43572007 | -1.349 |  |  | 8 | 4369 | 7486297 | 1.113 |  |  |
| hsa-miR-202* | 6 | 33 | 731 | 6 | 10 | 173 | 1.900 | * | 0.00017301/ NO | 8 | 115 | 3601 | -3.425 | *** | 0.000171233/ NO |
| hsa-miR-205 | 6 | 83 | 8305 | 6 | 111 | 4701 | -0.599 |  |  | 8 | 33 | 2319 | 1.225 |  |  |
| hsa-miR-206 | 6 | 1051 | 1667002 | 6 | 2531 | 6701857 | -1.253 |  |  | 8 | 983 | 749074 | 0.113 |  |  |
| hsa-miR-210 | 6 | 125 | 6583 | 6 | 213 | 15508 | -1.460 |  |  | 8 | 67 | 8735 | 1.229 |  |  |
| hsa-miR-211 | 6 | 188 | 12781 | 6 | 249 | 19076 | -0.843 |  |  | 8 | 148 | 13261 | 0.647 |  |  |
| hsa-miR-212 | 6 | 93 | 7446 | 6 | 232 | 40472 | -1.552 |  |  | 8 | 153 | 25569 | -0.894 |  |  |
| hsa-miR-213 | 6 | 63 | 1566 | 6 | 96 | 8686 | -0.801 |  |  | 8 | 93 | 5084 | -1.027 |  |  |
| hsa-miR-215 | 6 | 131 | 18817 | 6 | 197 | 32775 | -0.707 |  |  | 8 | 90 | 3155 | 0.693 |  |  |
| hsa-miR-216 | 6 | 127 | 10909 | 6 | 237 | 34328 | -1.267 |  |  | 8 | 151 | 14038 | -0.415 |  |  |
| hsa-miR-217 | 6 | 44 | 3502 | 6 | 79 | 4306 | -0.974 |  |  | 8 | 146 | 8825 | -2.501 | ** | 0.000188679/ NO |
| hsa-miR-218 | 6 | 145 | 10235 | 6 | 183 | 8759 | -0.678 |  |  | 8 | 44 | 2839 | 2.229 | ** | 0.000198413/ NO |
| hsa-miR-219 | 6 | 147 | 2870 | 6 | 46 | 753 | 4.111 | *** | 0.000160256/ NO | 8 | 69 | 6173 | 2.220 | ** | 0.000199203/ NO |
| hsa-miR-222 | 6 | 50 | 2600 | 6 | 56 | 7201 | -0.154 |  |  | 8 | 91 | 10471 | -0.989 |  |  |
| hsa-miR-223 | 6 | 409 | 44299 | 6 | 363 | 16724 | 0.461 |  |  | 8 | 387 | 67060 | 0.175 |  |  |
| hsa-miR-224 | 6 | 90 | 4125 | 6 | 137 | 12089 | -0.890 |  |  | 8 | 180 | 18782 | -1.633 |  |  |
| hsa-miR-299-5p | 6 | 412 | 43450 | 6 | 619 | 184744 | -1.066 |  |  | 8 | 508 | 148168 | -0.602 |  |  |
| hsa-miR-301 | 6 | 105 | 8777 | 6 | 170 | 39108 | -0.722 |  |  | 8 | 32 | 1613 | 1.794 | * | 0.000217391/ NO |
| hsa-miR-302a | 6 | 133 | 10318 | 6 | 204 | 39292 | -0.770 |  |  | 8 | 101 | 8199 | 0.629 |  |  |
| hsa-miR-302a* | 6 | 48 | 5512 | 6 | 5 | 160 | 1.385 |  |  | 8 | 141 | 9995 | -2.003 | * | 0.000210084/ NO |
| hsa-miR-302b | 6 | 25 | 3749 | 6 | 56 | 4887 | -0.824 |  |  | 8 | 37 | 1351 | -0.425 |  |  |
| hsa-miR-302c | 6 | 126 | 6152 | 6 | 239 | 27711 | -1.506 |  |  | 8 | 63 | 3368 | 1.649 |  |  |
| hsa-miR-302c* | 6 | 1433 | 3568024 | 6 | 2021 | 4116945 | -0.520 |  |  | 8 | 953 | 1118146 | 0.560 |  |  |
| hsa-miR-302d | 6 | 13 | 551 | 6 | 2 | 26 | 1.097 |  |  | 8 | 84 | 4527 | -2.769 | ** | 0.000181818/ NO |
| hsa-miR-30b | 6 | 38 | 965 | 6 | 83 | 9938 | -1.042 |  |  | 8 | 77 | 3439 | -1.581 |  |  |
| hsa-miR-30c | 6 | 18 | 1102 | 6 | 185 | 47600 | -1.861 | * | 0.000174216/ NO | 8 | 205 | 24499 | -3.282 | *** | 0.00017301/ NO |
| hsa-miR-30d | 6 | 208 | 32615 | 6 | 378 | 79098 | -1.250 |  |  | 8 | 234 | 25979 | -0.284 |  |  |
| hsa-miR-320 | 6 | 1493 | 2561458 | 6 | 2868 | 6140310 | -1.143 |  |  | 8 | 1195 | 841005 | 0.408 |  |  |
| hsa-miR-324-5p | 6 | 226 | 23839 | 6 | 376 | 173991 | -0.826 |  |  | 8 | 204 | 14334 | 0.285 |  |  |
| hsa-miR-325 | 6 | 269 | 48032 | 6 | 470 | 254196 | -0.892 |  |  | 8 | 63 | 8024 | 2.173 | * | 0.000202429/ NO |
| hsa-miR-326 | 6 | 541 | 200921 | 6 | 961 | 519220 | -1.212 |  |  | 8 | 310 | 35611 | 1.187 |  |  |
| hsa-miR-328 | 6 | 420 | 134620 | 6 | 514 | 127987 | -0.447 |  |  | 8 | 233 | 25491 | 1.171 |  |  |
| hsa-miR-329 | 6 | 26 | 1581 | 6 | 146 | 12032 | -2.520 | ** | 0.000161812/ NO | 8 | 53 | 4459 | -0.945 |  |  |
| hsa-miR-330 | 6 | 126 | 13056 | 6 | 162 | 26423 | -0.445 |  |  | 8 | 117 | 9932 | 0.154 |  |  |
| hsa-miR-331 | 6 | 74 | 6566 | 6 | 63 | 8464 | 0.223 |  |  | 8 | 21 | 3623 | 1.352 |  |  |
| hsa-miR-335 | 6 | 804 | 494040 | 6 | 1600 | 2363477 | -1.153 |  |  | 8 | 773 | 304616 | 0.091 |  |  |
| hsa-miR-337 | 6 | 72 | 9366 | 6 | 58 | 3713 | 0.293 |  |  | 8 | 117 | 8843 | -0.868 |  |  |
| hsa-miR-338 | 6 | 3 | 69 | 6 | 22 | 1922 | -1.016 |  |  | 8 | 102 | 4631 | -4.060 | *** | 0.000166667/ NO |
| hsa-miR-340 | 6 | 89 | 8809 | 6 | 78 | 7948 | 0.218 |  |  | 8 | 135 | 6117 | -0.960 |  |  |
| hsa-miR-346 | 6 | 313 | 53618 | 6 | 351 | 84287 | -0.255 |  |  | 8 | 199 | 22214 | 1.051 |  |  |
| hsa-miR-361 | 6 | 129 | 9815 | 6 | 180 | 27393 | -0.648 |  |  | 8 | 83 | 7530 | 0.894 |  |  |
| hsa-miR-374 | 6 | 169 | 14885 | 6 | 141 | 17854 | 0.376 |  |  | 8 | 119 | 13976 | 0.769 |  |  |
| hsa-miR-380-5p | 6 | 170 | 12562 | 6 | 223 | 28613 | -0.637 |  |  | 8 | 68 | 3744 | 2.016 | * | 0.000208333/ NO |
| hsa-miR-512-3p | 6 | 214 | 30687 | 6 | 432 | 114807 | -1.403 |  |  | 8 | 116 | 14339 | 1.183 |  |  |
| hsa-miR-515-3p | 6 | 64 | 10895 | 6 | 178 | 34939 | -1.309 |  |  | 8 | 144 | 10736 | -1.426 |  |  |
| hsa-miR-517a | 6 | 26 | 1281 | 6 | 70 | 6949 | -1.214 |  |  | 8 | 73 | 8687 | -1.304 |  |  |
| hsa-miR-517c | 6 | 179 | 8147 | 6 | 304 | 94352 | -0.955 |  |  | 8 | 222 | 21453 | -0.675 |  |  |
| hsa-miR-519a | 6 | 33 | 3163 | 6 | 99 | 20027 | -1.063 |  |  | 8 | 91 | 6586 | -1.582 |  |  |
| hsa-miR-519b | 6 | 100 | 7084 | 6 | 139 | 13412 | -0.665 |  |  | 8 | 122 | 9131 | -0.466 |  |  |
| hsa-miR-519c | 6 | 39 | 1633 | 6 | 74 | 7718 | -0.889 |  |  | 8 | 75 | 4444 | -1.254 |  |  |
| hsa-miR-519d | 6 | 128 | 44778 | 6 | 234 | 104033 | -0.678 |  |  | 8 | 118 | 10532 | 0.103 |  |  |
| hsa-miR-519e | 6 | 50 | 5216 | 6 | 38 | 1700 | 0.363 |  |  | 8 | 49 | 2176 | 0.025 |  |  |
| hsa-miR-521 | 6 | 21 | 676 | 6 | 39 | 1270 | -0.975 |  |  | 8 | 91 | 4892 | -2.598 | ** | 0.000185185/ NO |
| hsa-miR-525* | 6 | 21 | 812 | 6 | 17 | 1513 | 0.245 |  |  | 8 | 112 | 5704 | -3.128 | *** | 0.000176678/ NO |
| hsa-miR-106a | 6 | 327 | 51745 | 6 | 339 | 117387 | -0.068 |  |  | 8 | 117 | 6075 | 2.172 | * | 0.000203252/ NO |
| hsa-miR-106b | 6 | 291 | 48266 | 6 | 471 | 131655 | -1.044 |  |  | 8 | 189 | 11419 | 1.042 |  |  |
| hsa-miR-126* | 6 | 211 | 26596 | 6 | 275 | 40383 | -0.601 |  |  | 8 | 156 | 12823 | 0.708 |  |  |
| hsa-miR-17-5p | 6 | 211 | 29553 | 6 | 281 | 71755 | -0.535 |  |  | 8 | 59 | 2367 | 2.107 | * | 0.000204918/ NO |
| hsa-miR-19a | 6 | 155 | 26224 | 6 | 135 | 17772 | 0.241 |  |  | 8 | 135 | 3954 | 0.292 |  |  |
| hsa-miR-19b | 6 | 146 | 10377 | 6 | 286 | 31252 | -1.674 |  |  | 8 | 99 | 3097 | 1.037 |  |  |
| hsa-miR-204 | 6 | 62 | 10392 | 6 | 79 | 6470 | -0.322 |  |  | 8 | 100 | 6989 | -0.743 |  |  |
| hsa-miR-363 | 6 | 0 | 0 | 6 | 18 | 1903 | -1.000 |  |  | 8 | 67 | 2036 | -4.208 | *** | 0.000165017/ NO |
| hsa-miR-363* | 6 | 2658 | 7560790 | 6 | 4482 | 18103139 | -0.882 |  |  | 8 | 1195 | 496607 | 1.272 |  |  |
| hsa-miR-365 | 6 | 202 | 46387 | 6 | 301 | 95257 | -0.647 |  |  | 6 | 158 | 10253 | 0.458 |  |  |
| hsa-miR-367 | 6 | 221 | 30055 | 6 | 179 | 60029 | 0.344 |  |  | 8 | 32 | 825 | 2.649 | ** | 0.00018315/ NO |
| hsa-miR-371 | 6 | 248 | 71309 | 6 | 221 | 54295 | 0.184 |  |  | 8 | 8 | 128 | 2.199 | ** | 0.000201613/ NO |
| hsa-miR-372 | 6 | 6 | 145 | 6 | 6 | 176 | -0.030 |  |  | 8 | 62 | 2661 | -2.973 | ** | 0.000178571/ NO |
| hsa-miR-373 | 6 | 181 | 38951 | 6 | 154 | 28089 | 0.256 |  |  | 8 | 122 | 4912 | 0.707 |  |  |
| hsa-miR-375 | 6 | 3753 | 28106663 | 6 | 5009 | 43164352 | -0.365 |  |  | 8 | 898 | 1209222 | 1.298 |  |  |
| hsa-miR-376a* | 6 | 222 | 40269 | 6 | 259 | 42885 | -0.314 |  |  | 8 | 89 | 2402 | 1.590 |  |  |
| hsa-miR-379 | 6 | 257 | 87854 | 6 | 292 | 52198 | -0.227 |  |  | 8 | 177 | 7486 | 0.639 |  |  |
| hsa-miR-381 | 6 | 21 | 1127 | 6 | 34 | 1661 | -0.571 |  |  | 8 | 106 | 9248 | -2.315 | ** | 0.000194553/ NO |
| hsa-miR-382 | 6 | 165 | 77981 | 6 | 458 | 409222 | -1.028 |  |  | 8 | 98 | 7621 | 0.566 |  |  |
| hsa-miR-383 | 6 | 109 | 28127 | 6 | 290 | 144600 | -1.064 |  |  | 8 | 151 | 5895 | -0.573 |  |  |
| hsa-miR-384 | 6 | 147 | 26006 | 6 | 147 | 24153 | 0.003 |  |  | 8 | 100 | 3796 | 0.689 |  |  |
| hsa-miR-409-3p | 6 | 179 | 56264 | 6 | 188 | 26938 | -0.075 |  |  | 8 | 167 | 11193 | 0.113 |  |  |
| hsa-miR-412 | 6 | 202 | 88760 | 6 | 211 | 34052 | -0.059 |  |  | 8 | 111 | 5448 | 0.734 |  |  |
| hsa-miR-422a | 6 | 103 | 14110 | 6 | 192 | 34582 | -0.989 |  |  | 8 | 91 | 1874 | 0.234 |  |  |
| hsa-miR-422b | 6 | 97 | 24940 | 6 | 112 | 15292 | -0.181 |  |  | 8 | 45 | 2606 | 0.779 |  |  |
| hsa-miR-423 | 6 | 660 | 369855 | 6 | 762 | 295806 | -0.305 |  |  | 8 | 221 | 12233 | 1.746 |  |  |
| hsa-miR-424 | 6 | 135 | 16969 | 6 | 137 | 19524 | -0.022 |  |  | 8 | 81 | 1990 | 0.966 |  |  |
| hsa-miR-425 | 6 | 70 | 15151 | 6 | 295 | 71452 | -1.872 | * | 0.000173611/ NO | 8 | 158 | 15556 | -1.323 |  |  |
| hsa-miR-429 | 6 | 205 | 24500 | 6 | 292 | 69754 | -0.695 |  |  | 8 | 123 | 6800 | 1.165 |  |  |
| hsa-miR-432* | 6 | 119 | 20851 | 6 | 220 | 85776 | -0.757 |  |  | 8 | 85 | 5743 | 0.530 |  |  |
| hsa-miR-433 | 6 | 101 | 13804 | 6 | 295 | 184171 | -1.068 |  |  | 8 | 50 | 3177 | 0.986 |  |  |
| hsa-miR-449 | 6 | 223 | 31905 | 6 | 187 | 24237 | 0.370 |  |  | 8 | 47 | 1607 | 2.368 | ** | 0.000191571/ NO |
| hsa-miR-450 | 6 | 228 | 27256 | 6 | 217 | 22158 | 0.116 |  |  | 8 | 214 | 18234 | 0.168 |  |  |
| hsa-miR-451 | 6 | 0 | 0 | 6 | 40 | 2347 | -2.020 | * | 0.000170648/ NO | 8 | 53 | 2154 | -3.245 | *** | 0.000174825/ NO |
| hsa-miR-453 | 6 | 62 | 13099 | 6 | 187 | 45417 | -1.260 |  |  | 8 | 170 | 19499 | -1.589 |  |  |
| hsa-miR-455 | 6 | 26 | 1303 | 6 | 46 | 7831 | -0.514 |  |  | 8 | 193 | 6120 | -5.342 | *** | 0.000161812/ NO |
| hsa-miR-484 | 6 | 56 | 9142 | 6 | 78 | 2105 | -0.498 |  |  | 8 | 18 | 782 | 0.932 |  |  |
| hsa-miR-485-3p | 6 | 458 | 209155 | 6 | 487 | 309411 | -0.098 |  |  | 8 | 174 | 20661 | 1.469 |  |  |
| hsa-miR-486 | 6 | 81 | 18430 | 6 | 110 | 7869 | -0.425 |  |  | 8 | 61 | 2296 | 0.355 |  |  |
| hsa-miR-487a | 6 | 487 | 107091 | 6 | 636 | 235601 | -0.624 |  |  | 8 | 283 | 26997 | 1.400 |  |  |
| hsa-miR-487b | 6 | 31 | 2503 | 6 | 61 | 13682 | -0.569 |  |  | 8 | 159 | 16732 | -2.552 | ** | 0.000185874/ NO |
| hsa-miR-488 | 6 | 58 | 4828 | 6 | 77 | 12028 | -0.374 |  |  | 8 | 54 | 1623 | 0.100 |  |  |
| hsa-miR-490 | 6 | 458 | 271153 | 6 | 701 | 651392 | -0.620 |  |  | 8 | 142 | 8951 | 1.467 |  |  |
| hsa-miR-492 | 6 | 248 | 93407 | 6 | 358 | 187239 | -0.510 |  |  | 8 | 223 | 12037 | 0.193 |  |  |
| hsa-miR-493-3p | 6 | 199 | 52523 | 6 | 190 | 92370 | 0.058 |  |  | 8 | 130 | 8571 | 0.695 |  |  |
| hsa-miR-493-5p | 6 | 36 | 1687 | 6 | 30 | 3115 | 0.211 |  |  | 8 | 78 | 4335 | -1.474 |  |  |
| hsa-miR-494 | 6 | 3267 | 8900503 | 6 | 4286 | 10669488 | -0.565 |  |  | 8 | 1288 | 493898 | 1.592 |  |  |
| hsa-miR-495 | 6 | 262 | 40760 | 6 | 201 | 26904 | 0.571 |  |  | 8 | 176 | 21789 | 0.877 |  |  |
| hsa-miR-496 | 6 | 152 | 11539 | 6 | 99 | 7807 | 0.930 |  |  | 8 | 68 | 4284 | 1.708 |  |  |
| hsa-miR-518a | 6 | 49 | 5478 | 6 | 28 | 3781 | 0.550 |  |  | 8 | 75 | 4219 | -0.686 |  |  |
| hsa-miR-518b | 6 | 683 | 562486 | 6 | 950 | 632662 | -0.598 |  |  | 8 | 155 | 20398 | 1.701 |  |  |
| hsa-miR-518c | 6 | 0 | 0 | 6 | 14 | 435 | -1.613 |  |  | 6 | 88 | 4220 | -3.832 | *** | 0.000167224/ NO |
| hsa-miR-518d | 6 | 93 | 18881 | 6 | 157 | 45338 | -0.616 |  |  | 8 | 9 | 224 | 1.491 |  |  |
| hsa-miR-518e | 6 | 52 | 3640 | 6 | 57 | 8441 | -0.118 |  |  | 8 | 132 | 2398 | -2.665 | ** | 0.000182482/ NO |
| hsa-miR-518f | 6 | 171 | 19924 | 6 | 166 | 61001 | 0.042 |  |  | 8 | 88 | 3143 | 1.357 |  |  |
| hsa-miR-520a | 6 | 123 | 7552 | 6 | 28 | 2211 | 2.358 | ** | 0.000163934/ NO | 8 | 92 | 5587 | 0.705 |  |  |
| hsa-miR-520b | 6 | 182 | 54524 | 6 | 168 | 13734 | 0.136 |  |  | 8 | 110 | 3513 | 0.741 |  |  |
| hsa-miR-520c | 6 | 20 | 1102 | 6 | 39 | 2100 | -0.820 |  |  | 8 | 37 | 2036 | -0.817 |  |  |
| hsa-miR-520d | 6 | 123 | 32292 | 6 | 205 | 25200 | -0.839 |  |  | 8 | 163 | 7472 | -0.506 |  |  |
| hsa-miR-520e | 6 | 40 | 2559 | 6 | 10 | 198 | 1.398 |  |  | 8 | 125 | 12573 | -1.899 | * | 0.000213675/ NO |
| hsa-miR-520g | 6 | 3 | 22 | 6 | 3 | 34 | -0.109 |  |  | 8 | 34 | 868 | -2.914 | ** | 0.000179856/ NO |
| hsa-miR-520h | 6 | 22 | 1908 | 6 | 30 | 2032 | -0.323 |  |  | 8 | 22 | 2202 | -0.020 |  |  |
| hsa-miR-522 | 6 | 184 | 19321 | 6 | 199 | 25304 | -0.176 |  |  | 8 | 131 | 5413 | 0.847 |  |  |
| hsa-miR-523 | 6 | 186 | 28960 | 6 | 188 | 10120 | -0.024 |  |  | 8 | 23 | 432 | 2.334 | ** | 0.00019305/ NO |
| hsa-miR-125a | 6 | 322 | 135074 | 6 | 115 | 5981 | 1.353 |  |  | 8 | 179 | 16007 | 0.916 |  |  |
| hsa-miR-125b | 6 | 33 | 6195 | 6 | 33 | 2624 | -0.002 |  |  | 8 | 156 | 10997 | -2.503 | ** | 1. NO |
| hsa-miR-144 | 6 | 195 | 29699 | 6 | 128 | 16374 | 0.769 |  |  | 8 | 141 | 7645 | 0.702 |  |  |
| hsa-miR-148a | 6 | 177 | 22614 | 6 | 33 | 3243 | 2.200 | * | 0.000166113/ NO | 8 | 126 | 12910 | 0.697 |  |  |
| hsa-miR-148b | 6 | 108 | 25572 | 6 | 29 | 753 | 1.197 |  |  | 8 | 136 | 7899 | -0.393 |  |  |
| hsa-miR-149 | 6 | 457 | 65315 | 6 | 163 | 26943 | 2.375 | ** | 0.000162866/ NO | 8 | 188 | 25994 | 2.269 | ** | 0.00019685/ NO |
| hsa-miR-17-3p | 6 | 39 | 4392 | 6 | 24 | 1714 | 0.452 |  |  | 8 | 100 | 6944 | -1.527 |  |  |
| hsa-miR-181a | 6 | 98 | 37365 | 6 | 47 | 3199 | 0.617 |  |  | 8 | 102 | 4481 | -0.049 |  |  |
| hsa-miR-181b | 6 | 80 | 7862 | 6 | 78 | 4539 | 0.050 |  |  | 8 | 30 | 735 | 1.339 |  |  |
| hsa-miR-181c | 6 | 82 | 5820 | 6 | 14 | 873 | 2.032 | * | 0.000170068/ NO | 8 | 41 | 4616 | 1.056 |  |  |
| hsa-miR-181d | 6 | 150 | 55691 | 6 | 28 | 1535 | 1.251 |  |  | 8 | 55 | 2196 | 0.977 |  |  |
| hsa-miR-200a | 6 | 99 | 37164 | 6 | 13 | 423 | 1.079 |  |  | 8 | 143 | 11008 | -0.511 |  |  |
| hsa-miR-200b | 6 | 130 | 21570 | 6 | 71 | 12206 | 0.781 |  |  | 8 | 147 | 16311 | -0.233 |  |  |
| hsa-miR-200c | 6 | 0 | 0 | 6 | 3 | 28 | -1.470 |  |  | 8 | 74 | 2662 | -4.082 | *** | 0.000166113/ NO |
| hsa-miR-22 | 6 | 3 | 42 | 6 | 2 | 17 | 0.308 |  |  | 8 | 76 | 4269 | -3.153 | *** | 0.000176056/ NO |
| hsa-miR-221 | 6 | 0 | 0 | 6 | 7 | 60 | -2.067 | * | 0.000168919/ NO | 8 | 44 | 2641 | -2.441 | ** | 0.000190114/ NO |
| hsa-miR-26a | 6 | 213 | 25137 | 6 | 118 | 3573 | 1.387 |  |  | 8 | 135 | 7236 | 1.101 |  |  |
| hsa-miR-26b | 6 | 597 | 216314 | 6 | 265 | 41583 | 1.603 |  |  | 8 | 196 | 16832 | 2.056 | * | 0.000205761/ NO |
| hsa-miR-299-3p | 6 | 564 | 96067 | 6 | 357 | 100108 | 1.145 |  |  | 8 | 210 | 29016 | 2.520 | ** | 0.000186567/ NO |
| hsa-miR-29a | 6 | 218 | 37832 | 6 | 222 | 28132 | -0.035 |  |  | 8 | 96 | 4840 | 1.471 |  |  |
| hsa-miR-29b | 6 | 129 | 22638 | 6 | 8 | 307 | 1.952 | * | 0.000171233/ NO | 8 | 113 | 8456 | 0.225 |  |  |
| hsa-miR-29c | 6 | 323 | 51527 | 6 | 107 | 3266 | 2.252 | ** | 0.000165563/ NO | 8 | 32 | 1340 | 3.106 | *** | 0.000177305/ NO |
| hsa-miR-30e-3p | 6 | 435 | 146102 | 6 | 101 | 3730 | 2.119 | * | 0.000167224/ NO | 8 | 184 | 16957 | 1.545 |  |  |
| hsa-miR-342 | 6 | 97 | 8795 | 6 | 13 | 137 | 2.180 | * | 0.000166667/ NO | 8 | 94 | 5800 | 0.065 |  |  |
| hsa-miR-362 | 6 | 50 | 7598 | 6 | 13 | 286 | 1.016 |  |  | 8 | 113 | 7509 | -1.346 |  |  |
| hsa-miR-368 | 6 | 158 | 20478 | 6 | 47 | 1079 | 1.856 | * | 0.000174825/ NO | 8 | 115 | 9245 | 0.642 |  |  |
| hsa-miR-369-5p | 6 | 168 | 26513 | 6 | 96 | 5612 | 0.995 |  |  | 8 | 90 | 3937 | 1.125 |  |  |
| hsa-miR-370 | 6 | 920 | 772209 | 6 | 416 | 18548 | 1.389 |  |  | 8 | 341 | 90467 | 1.548 |  |  |
| hsa-miR-376a | 6 | 1245 | 865036 | 6 | 791 | 274208 | 1.042 |  |  | 8 | 589 | 181281 | 1.607 |  |  |
| hsa-miR-376b | 6 | 2986 | 3749894 | 6 | 1958 | 2680180 | 0.993 |  |  | 8 | 1191 | 675429 | 2.131 | * | 0.000204082/ NO |
| hsa-miR-377 | 6 | 12 | 708 | 6 | 3 | 39 | 0.845 |  |  | 8 | 107 | 6306 | -3.159 | *** | 0.000175439/ NO |
| hsa-miR-380-3p | 6 | 364 | 90116 | 6 | 56 | 463 | 2.502 | ** | 0.000162338/ NO | 8 | 62 | 3457 | 2.431 | ** | 0.00019084/ NO |
| hsa-miR-409-5p | 6 | 268 | 28443 | 6 | 113 | 13284 | 1.850 | * | 0.000175439/ NO | 8 | 176 | 16867 | 1.104 |  |  |
| hsa-miR-432 | 6 | 23 | 731 | 6 | 84 | 10895 | -1.385 |  |  | 8 | 93 | 9015 | -1.986 | * | 0.00021097/ NO |
| hsa-miR-483 | 6 | 92 | 15421 | 6 | 6 | 95 | 1.687 |  |  | 8 | 51 | 3114 | 0.757 |  |  |
| hsa-miR-497 | 6 | 496 | 155042 | 6 | 113 | 4750 | 2.345 | ** | 0.000164474/ NO | 8 | 160 | 9089 | 2.043 | * | 0.000206612/ NO |
| hsa-miR-498 | 6 | 7048 | 37208228 | 6 | 3685 | 1947712 | 1.316 |  |  | 8 | 1860 | 3925923 | 2.006 | * | 0.000209205/ NO |
| hsa-miR-499 | 6 | 273 | 38557 | 6 | 64 | 2766 | 2.523 | ** | 0.00016129/ NO | 8 | 83 | 4325 | 2.277 | ** | 0.000196078/ NO |
| hsa-miR-500 | 6 | 464 | 94944 | 6 | 231 | 11282 | 1.750 |  |  | 8 | 268 | 44934 | 1.335 |  |  |
| hsa-miR-501 | 6 | 426 | 56035 | 6 | 177 | 15123 | 2.289 | ** | 0.000165017/ NO | 8 | 69 | 2855 | 3.628 | *** | 0.000169492/ NO |
| hsa-miR-502 | 6 | 290 | 140352 | 6 | 82 | 5156 | 1.333 |  |  | 8 | 172 | 7909 | 0.757 |  |  |
| hsa-miR-503 | 6 | 2043 | 2951357 | 6 | 1250 | 705914 | 1.015 |  |  | 8 | 919 | 560631 | 1.499 |  |  |
| hsa-miR-504 | 6 | 448 | 107525 | 6 | 154 | 10377 | 2.096 | * | 0.000167785/ NO | 8 | 31 | 2291 | 3.091 | *** | 0.000177936/ NO |
| hsa-miR-505 | 6 | 63 | 12690 | 6 | 15 | 524 | 1.033 |  |  | 8 | 54 | 2416 | 0.192 |  |  |
| hsa-miR-506 | 6 | 5 | 130 | 6 | 0 | 0 | 1.000 |  |  | 8 | 31 | 1900 | -1.610 |  |  |
| hsa-miR-507 | 6 | 90 | 8585 | 6 | 75 | 2846 | 0.352 |  |  | 8 | 160 | 15084 | -1.212 |  |  |
| hsa-miR-508 | 6 | 1 | 7 | 6 | 4 | 115 | -0.736 |  |  | 8 | 17 | 621 | -1.752 |  |  |
| hsa-miR-509 | 6 | 283 | 142442 | 6 | 137 | 21663 | 0.883 |  |  | 8 | 141 | 26771 | 0.863 |  |  |
| hsa-miR-510 | 6 | 200 | 72931 | 6 | 64 | 248 | 1.230 |  |  | 8 | 110 | 4682 | 0.802 |  |  |
| hsa-miR-511 | 6 | 210 | 58228 | 6 | 48 | 1431 | 1.628 |  |  | 8 | 241 | 30638 | -0.262 |  |  |
| hsa-miR-512-5p | 6 | 263 | 30530 | 6 | 147 | 25137 | 1.210 |  |  | 8 | 199 | 17508 | 0.756 |  |  |
| hsa-miR-513 | 6 | 1252 | 1394933 | 6 | 869 | 289348 | 0.724 |  |  | 8 | 561 | 174571 | 1.371 |  |  |
| hsa-miR-514 | 6 | 22 | 606 | 6 | 53 | 4617 | -1.069 |  |  | 8 | 54 | 4237 | -1.301 |  |  |
| hsa-miR-516-3p | 6 | 183 | 18068 | 6 | 115 | 6536 | 1.071 |  |  | 8 | 46 | 3845 | 2.324 | ** | 0.000193798/ NO |
| hsa-miR-516-5p | 6 | 2087 | 2333579 | 6 | 974 | 406385 | 1.646 |  |  | 8 | 637 | 171830 | 2.263 | ** | 0.000197628/ NO |
| hsa-miR-518a-2* | 6 | 394 | 64044 | 6 | 219 | 18535 | 1.499 |  |  | 8 | 173 | 14555 | 1.985 | * | 0.000211864/ NO |
| hsa-miR-520a* | 6 | 116 | 43382 | 6 | 80 | 6305 | 0.392 |  |  | 8 | 160 | 12946 | -0.469 |  |  |
| hsa-miR-520d* | 6 | 191 | 15710 | 6 | 85 | 2043 | 1.947 | * | 0.000171821/ NO | 8 | 69 | 3425 | 2.207 | ** | 0.000200803/ NO |
| hsa-miR-526b* | 6 | 186 | 28371 | 6 | 40 | 2011 | 2.054 | * | 0.000169492/ NO | 8 | 74 | 6840 | 1.500 |  |  |
| hsa-miR-527 | 6 | 578 | 113185 | 6 | 346 | 31145 | 1.493 |  |  | 8 | 169 | 19210 | 2.807 | ** | 0.000181159/ NO |
| hsa-miR-539 | 6 | 32 | 1625 | 6 | 63 | 9223 | -0.726 |  |  | 8 | 18 | 904 | 0.750 |  |  |
| hsa-miR-542-3p | 6 | 6610 | 24965184 | 6 | 4354 | 9701161 | 0.938 |  |  | 8 | 2618 | 3121770 | 1.871 | * | 0.000214592/ NO |
| hsa-miR-542-5p | 6 | 28 | 4602 | 6 | 30 | 934 | -0.086 |  |  | 8 | 66 | 1892 | -1.219 |  |  |
| hsa-miR-544 | 6 | 187 | 51577 | 6 | 41 | 2792 | 1.540 |  |  | 8 | 52 | 807 | 1.444 |  |  |
| hsa-miR-545 | 6 | 66 | 8988 | 6 | 75 | 17499 | -0.140 |  |  | 8 | 6 | 130 | 1.533 |  |  |
| hsa-miR-9* | 6 | 79 | 11167 | 6 | 9 | 367 | 1.604 |  |  | 8 | 62 | 2673 | 0.373 |  |  |
|  |  |  |  |  |  |  |  |  |  |  |  |  |  |  |  |
