# Supplementary material for: Differential Expression of Exosomal microRNAs in Prefrontal Cortices of Schizophrenia and Bipolar Disorder Patients
Source: PLoS One. 2013 Jan 30;8(1):e48814. doi: 10.1371/journal.pone.0048814 (PMC3559697; doi:10.1371/journal.pone.0048814)
Supplement: Table S2 — Quantities of exosome-derived miRNA from the analyzed cases for Luminex assay, before and after NCode amplification. (DOCX) [file pone.0048814.s005.docx]

|  | miRNA input for NCODE amplification (ng) | miRNA amount after NCODE amplification (ng/ul*) |
| --- | --- | --- |
| C1 | 25 | 1328 |
| C2 | 25 | 1675 |
| C3 | 25 | 6049 |
| C4 | 1.8 | 2540 |
| C5 | 14 | 1938 |
| C6 | 25 | 1584 |
| BD1 | 25 | 18922 |
| BD2 | 25 | 37538 |
| BD3 | 20 | 192 |
| BD4 | 25 | 1971 |
| BD4 | 25 | 1849 |
| BD6 | 25 | 1429 |
| SZ1 | 25 | 4151 |
| SZ2 | 25 | 4148 |
| SZ3 | 25 | 4459 |
| SZ4 | 25 | 2755 |
| SZ5 | 2 | 1277 |
| SZ6 | 25 | 2742 |
| SZ7 | 25 | 6461 |
| SZ8 | 25 | 509 |

* final volume12 ul
