# Supplementary material for: Differential Expression of Exosomal microRNAs in Prefrontal Cortices of Schizophrenia and Bipolar Disorder Patients
Source: PLoS One. 2013 Jan 30;8(1):e48814. doi: 10.1371/journal.pone.0048814 (PMC3559697; doi:10.1371/journal.pone.0048814)
Supplement: Table S3 — Wilcoxon non-parametric test and fold change for top BD hits as means for statistical verification. 12 top-ranked miRNAs according to SAM in Table 3, with q-values lower than 15%, ending with miR-526b* - the last miRNA to have significantly changed expression in BD according to this test. miRNAs 219, 380–3p, 148a, 520a, and 526b* (p-values in bold) have significantly changed expression in BD cases in comparison to controls by this analysis. The expression of miR-29c is not significantly different in BD cases according to Wilcoxon test (p-value 0.0649; see Discussion). (DOCX) [file pone.0048814.s006.docx]

miRNA Fold Change in BD Wilcoxon rank-sum test

(p-values)

hsa-miR-219 -3.174775745 **0.0043**

hsa-miR-380-3p -6.446648952 **0.0411**

hsa-miR-499 -4.282320969 0.0931

hsa-miR-497 -4.382718595 0.0649

hsa-miR-149 -2.810465551 0.132

hsa-miR-501 -2.410634103 0.132

hsa-miR-29c -3.004718885 0.0649

hsa-miR-30e-3p -4.328156707 0.0777

hsa-miR-504 -2.901867898 0.132

hsa-miR-148a -5.395464461 **0.0411**

hsa-miR-520a -4.426740312 **0.026**

hsa-miR-526b* -4.623685461 **0.026**
