# Supplementary material for: Differential Expression of Exosomal microRNAs in Prefrontal Cortices of Schizophrenia and Bipolar Disorder Patients
Source: PLoS One. 2013 Jan 30;8(1):e48814. doi: 10.1371/journal.pone.0048814 (PMC3559697; doi:10.1371/journal.pone.0048814)
Supplement: Table S4 — Phenotype traits (tobacco, alcohol, and recreational drug use) of analyzed cases. (DOCX) [file pone.0048814.s007.docx]

| Case | Tobacco | Alcohol abuse | Drug abuse |
| --- | --- | --- | --- |
| SZ1 | yes | remote | no |
| SZ2 | yes | remote | no |
| SZ3 | yes | no | no |
| SZ4 | yes | remote | no |
| SZ5 | yes | no | no |
| SZ6 | yes | no | no |
| SZ7 | ? | ?, no at death | ?, no at death |
| SZ8 | ? | ?, no at death | ?, no at death |
| BD1 | yes | no | no |
| BD2 | yes | yes | no |
| BD3 | yes | yes | no |
| BD4 | ? | ?, no at death | ?, no at death |
| BD5 | ? | yes | ?, no at death |
| BD6 | ? | remote | ?, no at death |
| BD7 | remote | remote | no |
| BD8 | remote | no | no |
| BD9 | ? | ?, no at death | ?, no at death |
| C1 | ? | ?, no at death | ?, no at death |
| C2 | ? | ?, no at death | ?, no at death |
| C3 | ? | ?, no at death | ?, no at death |
| C4 | ? | ?, no at death | ?, no at death |
| C5 | ? | ?, no at death | ?, no at death |
| C6 | ? | ?, no at death | ?, no at death |
| C7 | ? | ?, no at death | ?, no at death |
| C8 | ? | ?, no at death | ?, no at death |
| C9 | ? | ?, no at death | ?, no at death |
| C10 | yes | no | no |
| C11 | ? | ? | ? |
| C12 | no | no | no |
| C13 | no | no | no |
