# Supplementary material for: Differential Expression of Exosomal microRNAs in Prefrontal Cortices of Schizophrenia and Bipolar Disorder Patients
Source: PLoS One. 2013 Jan 30;8(1):e48814. doi: 10.1371/journal.pone.0048814 (PMC3559697; doi:10.1371/journal.pone.0048814)
Supplement: Table S5 — An ANOVA analysis of differences in age between C, BD, and SZ groups of cases. No significant difference in age between the groups exists. (DOCX) [file pone.0048814.s008.docx]

|  | C | BD | SZ |
| --- | --- | --- | --- |
| average age | 64.15384615 | 66.22222222 | 69.875 |
| variance of age | 327.974359 | 394.1944444 | 254.9821429 |
| ANOVA |  | 0.130329571 | 1.179099342 |
| p-value |  | 0.721875883 | 0.291124791 |
